# A well-defined, versatile photoinitiator (salen)Co–CO_2_CH_3_ for visible light-initiated living/controlled radical polymerization†
†Electronic supplementary information (ESI) available: Experimental details, characterization of (salen)Co–CO_2_CH_3_, Fig. S1–S20. CCDC 1031130. For ESI and crystallographic data in CIF or other electronic format see DOI: 10.1039/c5sc00477b



**DOI:** 10.1039/c5sc00477b

**Published:** 2015-03-05

**Authors:** Yaguang Zhao, Mengmeng Yu, Shuailin Zhang, Zhenqiang Wu, Yuchu Liu, Chi-How Peng, Xuefeng Fu

**Affiliations:** a Beijing National Laboratory for Molecular Sciences , State Key Lab of Rare Earth Materials Chemistry and Applications , College of Chemistry and Molecular Engineering , Peking University , Beijing , 100871 , China . Email: fuxf@pku.edu.cn ; Fax: +86 10 6275 1708; b Department of Chemistry and Frontier Research Center on Fundamental and Applied Sciences of Matters , National Tsing-Hua University , Hsinchu , 30013 , Taiwan

## Abstract

The control of the polymerization of a wide range of monomers under mild conditions by a single catalyst remains a major challenge in polymer science. We report a versatile, well-defined organocobalt salen complex to control living radical polymerization of different categories of monomers, including acrylates, acrylamides and vinyl acetate, under visible light irradiation at ambient temperature. Both household light and sunlight were effectively applied in the synthesis of polymers with controlled molecular weights and narrow polydispersities. Narrowly dispersed block copolymers (*M*_w_/*M*_n_ < 1.2) were obtained under various conditions. The structures of the polymers were analyzed by ^1^H NMR, ^2^D NMR, ^13^C NMR, GPC, MALDI-TOF-MS and isotopic labeling experiments, which showed that the ω and α ends of the polymer chains were capped with (salen)Co and –CO_2_CH_3_ segments, respectively, from the photoinitiator (salen)Co–CO_2_CH_3_. The ω end was easily functionalized through oxygen insertion followed by hydrolysis from ^18^O_2_ to –^18^OH. This robust system can proceed without any additives, and offers a versatile and green way to produce well-defined homo and block copolymers.

## Abstract 2

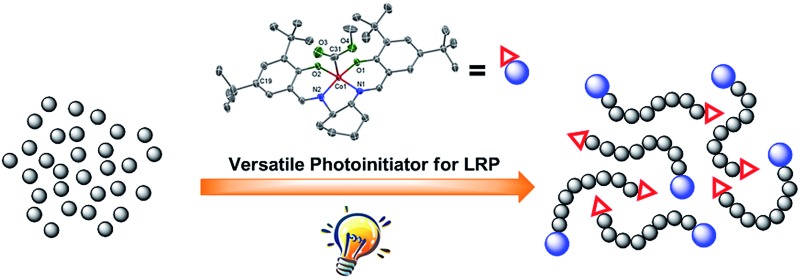
A well-defined organocobalt salen complex (salen)Co–CO_2_CH_3_ is used as a versatile photoinitiator for visible light-initiated living radical polymerization.

## Introduction

Precise control over macromolecular compositions and architectures has long been a challenge in polymer science. The advent of living radical polymerization (LRP) has significantly contributed to this field over the last two decades, and gives the possibility to obtain well-defined functional polymeric materials with precisely controlled structures and desired morphologies. So far, based on various strategies to regulate the equilibrium between dormant and active species in LRP, many powerful living radical polymerization technologies have been developed, such as nitroxide-mediated radical polymerization (NMP),^1^ atom transfer radical polymerization (ATRP),^2^ reversible addition–fragmentation chain transfer polymerization (RAFT),^3^ organoiodine-mediated radical polymerization (IRP),^4^ and organometallic-mediated radical polymerization (OMRP),^5^ *etc.*

Recently, the modulation of external stimuli to control radical polymerization through regulation of the activation and deactivation processes has been employed as an effective method to synthesize polymers with controlled molecular weights, narrow polydispersities and end group functionality.^6^ Of the various physical and chemical stimuli used, including heat, metal catalysts, and electrochemical,^7^ mechanical^6*a*^ and photochemical stimuli,^6^ light-induced LRPs^8–10^ are receiving growing attention due to their abundance, renewability and simple operation process. Moreover, light-induced LRPs provide opportunities to prepare novel materials through spatial and temporal control of the polymerization.^11^ Development of a versatile LRP catalyst suitable for a broader range of monomers, including both conjugated monomers (acrylates, acrylamides) and unconjugated monomers (vinyl esters), under mild conditions, would be desirable and represent a breakthrough in polymeric material synthesis.^3d,12^

Very recently, we reported the visible light-induced LRP of acrylates and acrylamides mediated by organocobalt porphyrins at room temperature, based on the photo-induced reversible cleavage of the Co–C bond.^13^ Structurally closely-related organocobalt salen (salen is a common abbreviation for N_2_O_2_ bis-Schiff base bis-phenolates) complexes also have relatively weak Co–C bonds. These complexes are supposed to undergo a similar photolysis process to generate an organic radical that could initiate polymerization and salen cobalt(ii), which functions as a persistent metal-centered radical to control polymerization. Compared with cobalt porphyrin, cobalt salen is more desirable for industrial applications due to its convenient synthesis, allowing facile adjustment of electronic and steric effects.^14^ Thus, cobalt salen complexes appear to be an ideal candidate to achieve photo-induced LRP. In fact, cobalt salen complexes have been widely used in the hydrolytic kinetic resolution^15^ and enantioselective ring opening polymerization of epoxides.^16^ However, they have rarely been applied to catalyze living radical polymerization until recently.^17^ To the best of our knowledge, there is no report on the success of photo-initiated LRP mediated by a cobalt salen complex.

**Scheme 1:**
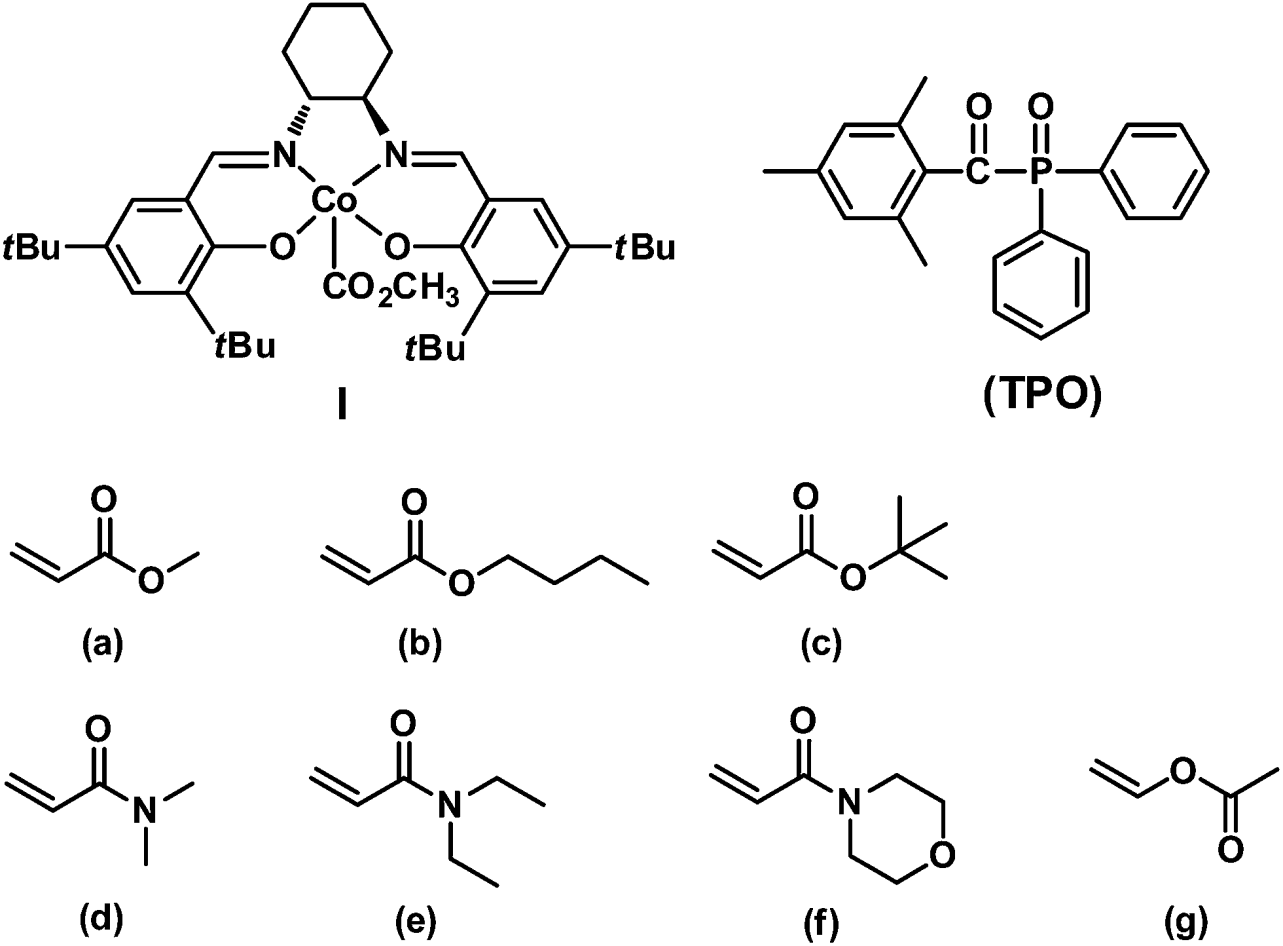
Structures of (salen)Co–CO_2_CH_3_ **I** and TPO and a list of monomers investigated in this study: (a) methyl acrylate (MA), (b) *n*-butyl acrylate (*n*BA), (c) *tert*-butyl acrylate (*t*BA), (d) *N*,*N*-dimethylacrylamide (DMA), (e) *N*,*N*-diethylacrylamide (DEA), (f) *N*-acryloylmorpholine (AMO), (g) vinyl acetate (VAc).

In this article, we report the first example of visible light-initiated LRP of a diverse range of monomers (acrylates, acrylamides and vinyl acetate) (Scheme 1) using a novel, well-defined methoxylcarbonyl–cobalt salen complex [(salen)Co–CO_2_CH_3_, **I**, salen = *N*,*N*′-bis(3,5-di-*tert*-butylsalicylidene)-1,2-cyclohexanediamine]. Complex **I** functions as both initiator and mediator to control the polymerization without any additives, under visible light or sunlight irradiation. Furthermore, the addition of the traditional photoinitiator 2,4,6-trimethylbenzoyl diphenylphosphine oxide (TPO) (Scheme 1) dramatically increases the polymerization efficiency, while maintaining good controllability. Visible light is essential in the initiation step, albeit dispensable during the chain propagation process, which differs from most photo-LRP processes that require continuous irradiation after the initiation step. The polymer structures were carefully investigated and were demonstrated to maintain the starting organocobalt salen segments at the chain ends. Well-controlled diblock copolymers were also easily prepared under mild conditions.

## Results and discussion

### Synthesis and characterization of (salen)Co–CO_2_CH_3_

**Fig. 1:**
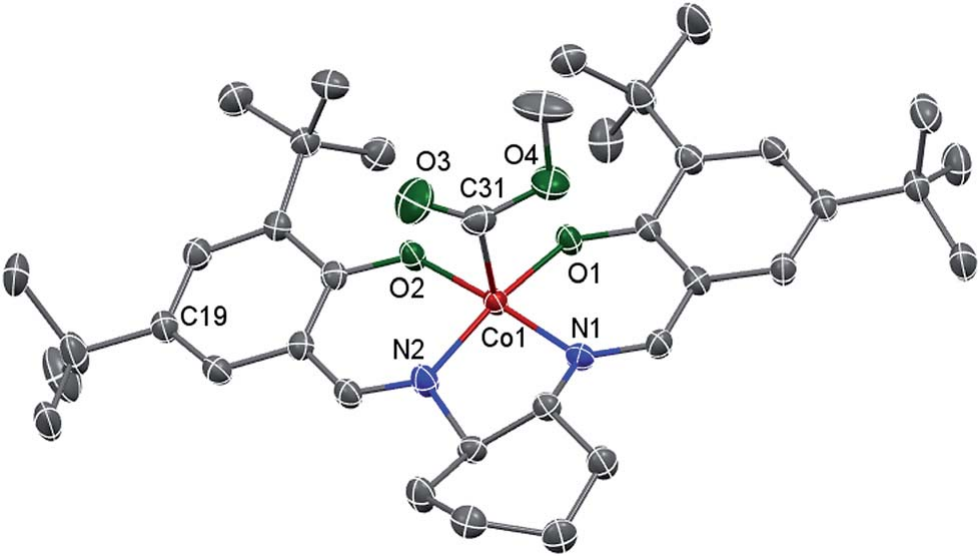
X-ray crystal structure of **I** shown with 50% thermal ellipsoids. One disordered *tert*-butyl group (at the C19 atom), cyclohexane and H atoms are omitted for clarity. Selected bond lengths [Å] and angles [°]: Co–O1 1.858(2), Co–O2 1.878(2), Co–N1 1.872(3), Co–N2 1.872(3), Co–C31 1.895(4); O1–Co–O2 85.32(9), O1–Co–N1 93.84(10), N2–Co–O2 93.96(10), N2–Co–N1 85.37(11), O1–Co–C31 96.95(13), O2–Co–C31 92.32(13), N1–Co–C31 94.67(14), N2–Co–C31 95.40(14).

**Fig. 2:**
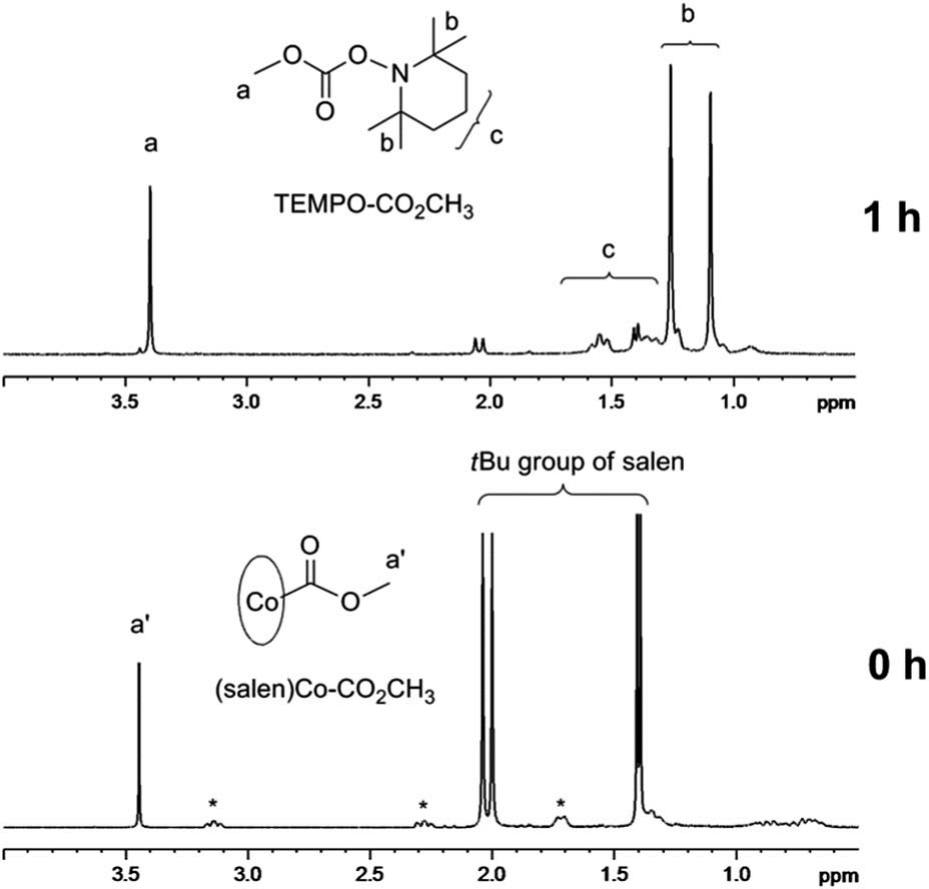
^1^H NMR spectra (in the range of 0.5–4.0 ppm) of (salen)Co–CO_2_CH_3_ **I** (5.0 mM) and TEMPO (50 mM) in benzene-*d*_6_ under visible light irradiation (*I* = 3 mW cm^–2^ at 420–780 nm wavelength) at room temperature for 1 h (*salen ligand in **I**, see ESI, Fig. 2S†).

**Fig. 3:**
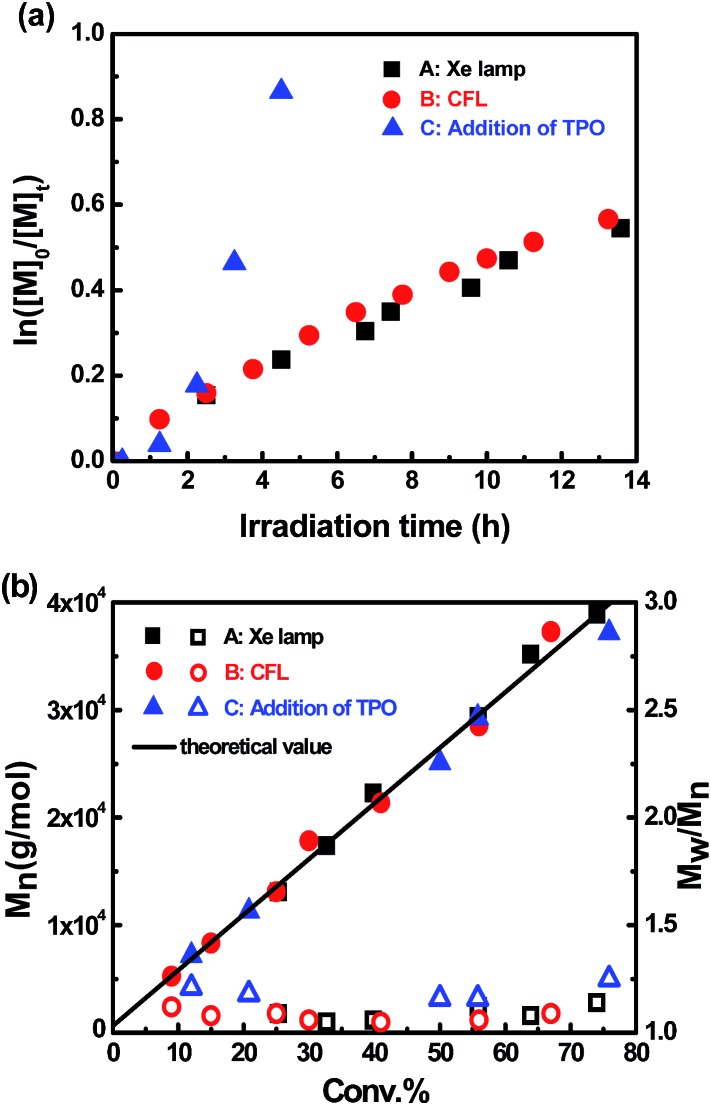
(a) Kinetic plots for polymerization of MA in benzene at ambient temperature with [MA]_0_ = 1.0 M, [MA]_0_/[**I**]_0_ = 600/1. (A): *t* = 13.6 h, conv. = 42%, *M*_n,GPC_ = 20 300, *M*_w_/*M*_n_ = 1.07. (B): *t* = 13.5 h, conv. = 45%, *M*_n,GPC_ = 26 100, *M*_w_/*M*_n_ = 1.06. (C): addition of 1 equivalent of TPO, *t* = 4.5 h, conv. = 58%, *M*_n,GPC_ = 28 500, *M*_w_/*M*_n_ = 1.12. (b) Evolution of molar mass and polydispersity with conversion under different conditions.

Living radical polymerization mediated by cobalt complexes often requires induction time to convert the cobalt species to trivalent organocobalt complexes, which are the actual mediators for the polymerization.^18^ Unfortunately, living radical polymerizations using a well-defined molecular organocobalt(iii) salen complex as the sole radical source are rare because such complexes are sensitive to air and light and are difficult to purify.^17a,19^ Here, we synthesized a typical trivalent (salen)Co–CO_2_CH_3_ (**I**) complex from the one-pot reaction of commercial (salen)Co(ii), methanol and CO by using Oxone as an oxidant. Compound **I** was obtained as a dark green solid and characterized using ^1^H NMR, ^13^C NMR, ESI-MS and FT-IR (ESI, Fig. 1S–3S†). This compound exhibits a strong CO stretching absorption at 1680 cm^–1^, which is similar to the analogous (TMP)Co–CO_2_CH_3_ (1695 cm^–1^, TMP refers to tetramesityl porphyrin).^13*b*^ The single crystal structure of this diamagnetic complex, **I**, is shown in Fig. 1. As with the reported similar cobalt alkyl complexes,^17a,20^ the cobalt atom is five-coordinated with a distorted square pyramidal geometry. The Co–O and Co–N bond lengths of *ca.* 1.868 and 1.872 Å are similar to those found in the analogue (saloph)Co-*i*C_3_H_7_ (saloph = dianion of disalicylidene-*o*-phenylenediamine, 1.872(5) and 1.879(5) Å, respectively).^20^ The Co–C bond length is 1.895 Å, indicating a stronger Co–C bond compared with that reported for (saloph)Co-*i*C_3_H_7_ (2.031(8) Å).^20^

### Photolysis of (salen)Co–CO_2_CH_3_

**Fig. 4:**
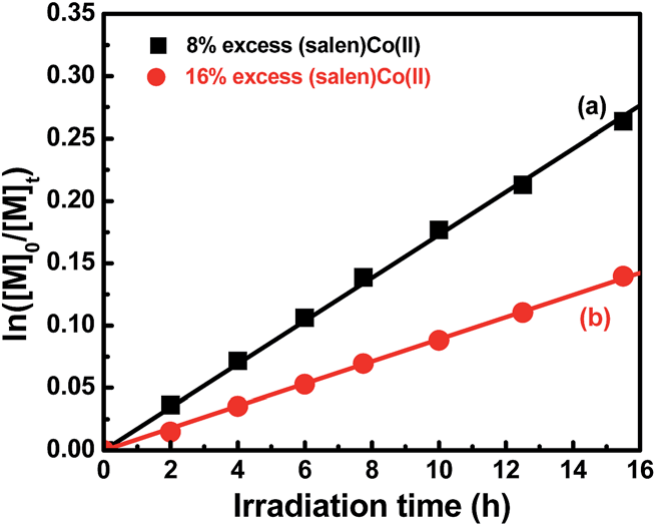
Kinetic plots for the polymerization of MA with an excess amount of (salen)Co(ii). [MA]_0_ = 1.0 M, [MA]_0_/[**I**]_0_ = 600/1. 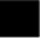 8% (salen)Co(ii) added, conv. = 23%, *M*_n,th_ = 12 500, *M*_n,GPC_ = 11 600, *M*_w_/*M*_n_ = 1.08. 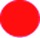 16% (salen)Co(ii) added, conv. = 13%, *M*_n,th_ = 7380, *M*_n,GPC_ = 7370, *M*_w_/*M*_n_ = 1.09.

**Fig. 5:**
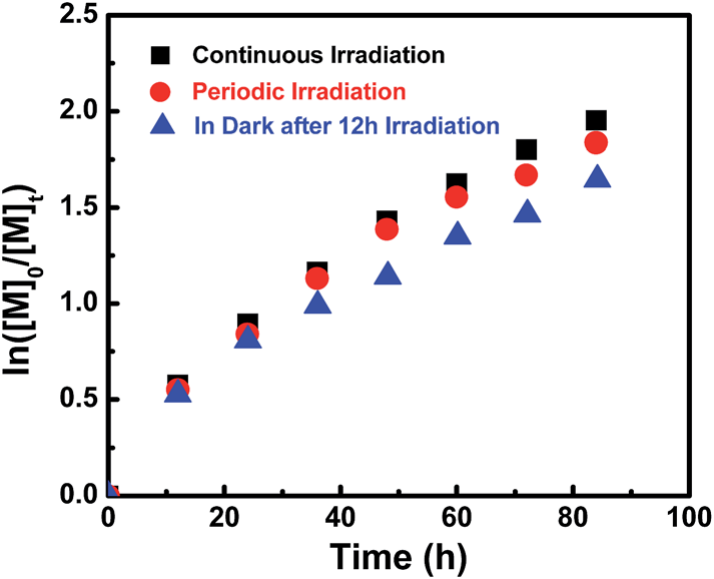
Kinetic plots for the polymerization of MA in benzene at ambient temperature under variable conditions. [MA]_0_ = 1.0 M, [MA]_0_/[**I**]_0_ = 200/1. 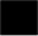 conv. = 87%, *M*_n,th_ = 15 600, *M*_n,GPC_ = 18 200, *M*_w_/*M*_n_ = 1.11. 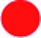 conv. = 85%, *M*_n,th_ = 15 300, *M*_n,GPC_ = 17 900, *M*_w_/*M*_n_ = 1.09. 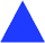 conv. = 81%, *M*_n,th_ = 14 600, *M*_n,GPC_ = 17 700, *M*_w_/*M*_n_ = 1.10.

**Fig. 6:**
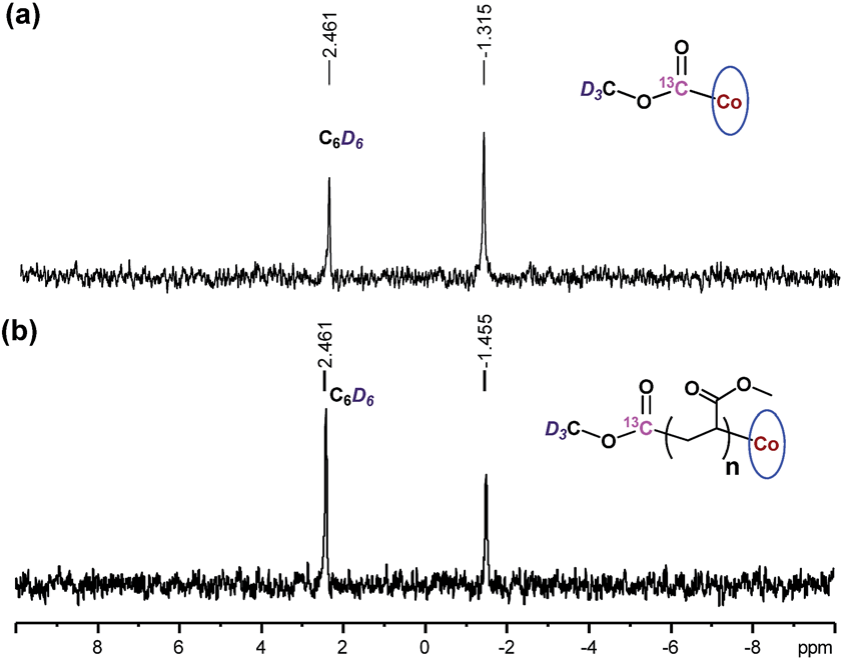
^2^D NMR spectra of benzene solutions of (salen)Co–^13^CO_2_CD_3,_ and PMA synthesized by photo-LRP mediated by (salen)Co–^13^CO_2_CD_3_ (*M*_n,GPC_ = 9060, *M*_w_/*M*_n_ = 1.08, *M*_n,th_ = 9125).

**Fig. 7:**
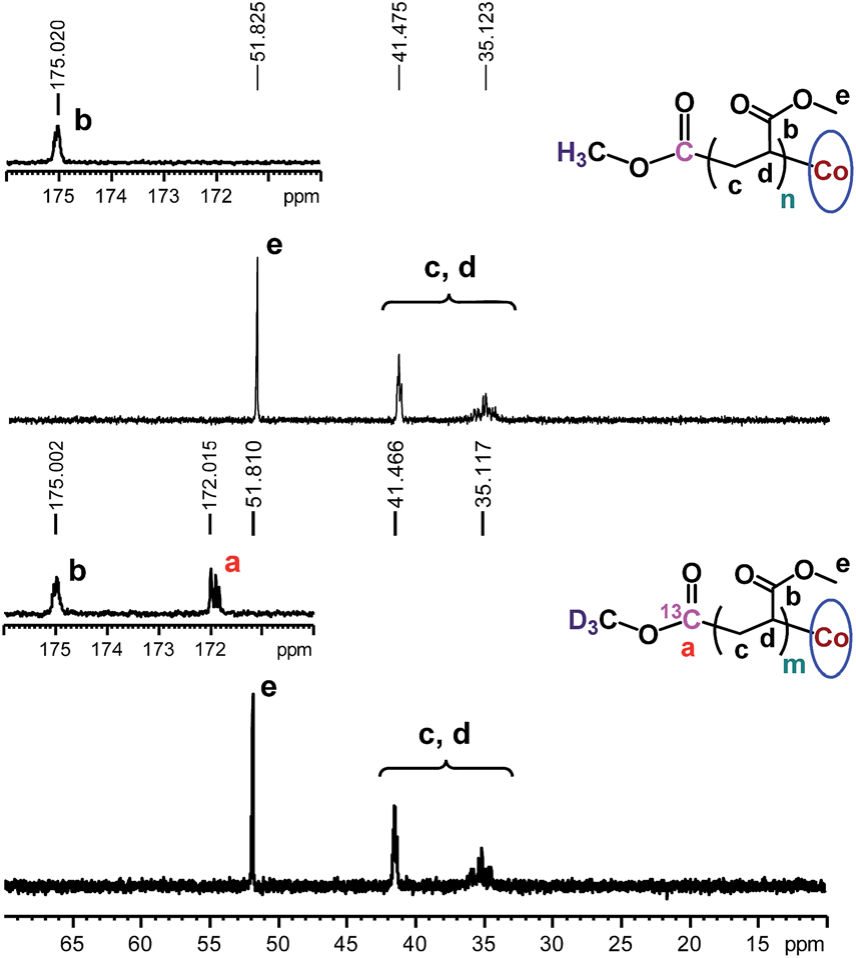
^13^C NMR spectra of PMA synthesized by photo-LRP mediated by (salen)Co–CO_2_CH_3_ (*M*_n,GPC_ = 7370, *M*_w_/*M*_n_ = 1.09) and (salen)Co–^13^CO_2_CD_3_ (*M*_n,GPC_ = 9060, *M*_w_/*M*_n_ = 1.08). CDCl_3_ was used as the solvent.

**Fig. 8:**
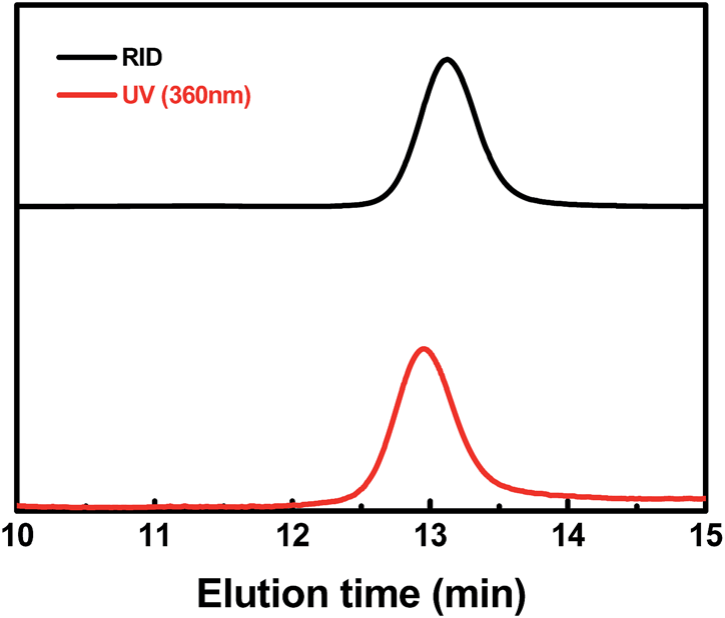
Gel permeation chromatography (GPC) traces of the PMA produced by photo-LRP mediated by **I** (*M*_n,th_ = 21 800, *M*_n,GPC_ = 21 400, *M*_w_/*M*_n_ = 1.05). The black line indicates the refractive index detection trace, and the red line indicates the UV-visible (360 nm) detection trace.

(Salen)Co–CO_2_CH_3_ shows a broad absorption band from 300 nm to 700 nm with the peak values around 333 nm and 625 nm (ESI, Fig. 4S†). To confirm the possibility of using (salen)Co–CO_2_CH_3_ as the photoinitiator, we carried out a typical radical trapping experiment under visible light irradiation (Fig. 2). The light source was a 500 W xenon lamp equipped with a 420–780 nm filter, with a light intensity of 3 mW cm^–2^ at the sample position. The benzene-*d*_6_ solution of (salen)Co–CO_2_CH_3_ (5.0 mM) and 2,2,6,6-tetramethylpiperidinyl-1-oxy (TEMPO) (50 mM) as the radical trap was irradiated for 1 h at ambient temperature. The photolysis occurred accompanied by a color change of the solution from dark green to red, indicating the formation of (salen)Co(ii) (ESI, Fig. 5S and 6S†). Fig. 2 shows the ^1^H NMR spectrum before and after visible light irradiation. Sharp signals in the region of 1.30–2.10 ppm corresponding to the *tert*-butyl group of **I** disappeared after irradiation for 1 h, which suggests the cleavage of the Co–C bond in (salen)Co–CO_2_CH_3_. Moreover, the resulting CH_3_CO_2_˙ radical was trapped by excess TEMPO to form TEMPO–CO_2_CH_3_ with a yield of 95%.^21^ This product is evidenced by the chemical shifts at *δ* = 1.05–1.30 ppm corresponding to the methyl protons in TEMPO–CO_2_CH_3_ (Fig. 2). The formation of TEMPO–CO_2_CH_3_ was also confirmed by ESI-MS (Fig. 7S†). Simultaneously, broad peaks at –0.49 ppm and downfield in the ^1^H NMR (ESI, Fig. 8S†) after photolysis clearly indicate the formation of the corresponding (salen)Co(ii).

### Visible light-initiated LRP mediated by (salen)Co–CO_2_CH_3_

The polymerization of different acrylates and acrylamides using **I** as both initiator and mediator at ambient temperature was tested. Three different light sources were used, including a 500 W xenon lamp, a commercial household compact fluorescent lamp and direct use of sunlight (Table 1). By using the xenon lamp or the fluorescent lamp, irradiation of the dark green solution of complex **I** with acrylates in benzene at room temperature yielded the corresponding polyacrylates, as evidenced by a color change to yellow (ESI, Fig. 5S†). The polymerizations were all performed with excellent control over molecular weight, with very narrow polydispersities along with the monomer conversions (Table 1, entries 1–6). The experimental molecular weights were all very close to the theoretical values and the GPC traces were monomodal and symmetrical. Thus, the visible light-initiated polymerization of acrylates mediated by **I** demonstrated very good control capability over acrylates using a xenon lamp or household light sources. Following the successful polymerization of acrylates, polymerization of acrylamides including DMA, DEA and AMO with **I** also resulted in polyacrylamides with narrow polydispersities (Table 1, entries 9–15). To avoid gel formation due to high viscosity, the ratio of AMO to **I** was reduced to 200, and this gave excellent control over molecular weight. Furthermore, direct use of sunlight without filtering has rarely been found in photo-LRP examples.^8b,c,9j,10f,13b,22^ However, in our system, the polymerization of DEA with sunlight irradiation gave PDEA, with a predetermined molecular weight and narrow polydispersity (Table 1, entry 13). To confirm that polymerization was induced only by **I** under light, control experiments in the absence of light for the polymerization of *t*BA and DMA were conducted (Table 1, entries 7–8). No polymers were detected in each case and no color change in solution was observed, indicating no polymerization occurred. Thus, visible light was essential for successful polymerization. To the best of our knowledge, this is the first example of a well-defined five-coordinate cobalt salen complex being used to give well-controlled polymerization under visible light irradiation at room temperature.

**Table 1:** Visible light-initiated LRP of acrylates and acrylamides using **I** as both initiator and mediator at ambient temperature^*a*^

Entry	Monomer (equiv.)	Condition^*b*^	*t* (h)	Conv.^*c*^ (%)	*M*_n,th_ ^*d*^	*M*_n,GPC_ ^*e*^	*M*_w_/*M*_n_ ^*e*^
1	MA (600)	Xe lamp	92	74	38 700	38 900	1.14
2	MA (600)	CFL	72	67	35 300	37 300	1.09
3	*n*BA (600)	Xe lamp	72	81	62 900	64 200	1.23
4	*n*BA (600)	CFL	72	88	68 300	71 700	1.23
5	*t*BA (600)	Xe lamp	24	73	56 800	55 000	1.11
6	*t*BA (600)	CFL	24	73	56 800	57 900	1.15
7^*f*^	*t*BA (600)	Dark	36	0	—	—	—
8^*f*^	DMA (600)	Dark	48	0	—	—	—
9	DMA (600)	Xe lamp	12	76	45 800	41 200	1.16
10	DMA (600)	CFL	12	79	47 600	43 100	1.16
11	DEA (600)	Xe lamp	4	71	54 100	57 200	1.22
12	DEA (600)	CFL	4	70	54 800	55 900	1.19
13	DEA (600)	Sunlight	4	42	32 400	35 600	1.23
14	AMO (200)	Xe lamp	12	84	24 400	20 700	1.12
15	AMO (200)	CFL	12	88	25 400	21 100	1.13

^*a*^[M]_0_ = 1.0 M in benzene-*d*_6_.

^*b*^A 500 W Xe lamp was used as the light source with a 420–780 nm filter, and the light intensity was 3 mW cm^–2^; a household CFL (compact fluorescent lamp, 27 W) was used as the light source, the light intensity was 3–5 mW cm^–2^ at the sample position; sunlight was used as the light source, and the sample was placed in a water bath at ambient temperature.

^*c*^The monomer conversion was determined based on the ^1^H NMR spectra.

^*d*^ *M*_n,th_ = *M*_w(_ **_I_**_)_ + *M*_w(Monomer)_ × ratio × conv. (%), where ratio refers to the ratio of monomer to **I**.

^*e*^Determined using gel permeation chromatography in DMF, calibrated against poly(methyl methacrylate) standards.

^*f*^Sample was protected from light with aluminum foil.

### Visible light-initiated LRP by addition of TPO

Although the polymerizations by direct irradiation of **I** and different acrylates or acrylamides all gave promising results, the polymerization rates were rather slow. Polymerization of MA required 92 h irradiation to achieve 74% conversion. Switching from reversible termination (RT) to degenerate transfer (DT), by addition of external radicals, could dramatically increase the rate of polymerization.^18b,23^ Thus, addition of a traditional photoinitiator to generate excess radicals would be helpful to improve the polymerization efficiency. Indeed, the photopolymerization of MA (600 equiv.) with organocobalt complex **I** in the presence of TPO (1 equiv.) reached 76% conversion within 8 h at ambient temperature, while 92 h were required for similar monomer conversion without TPO (Table 1, entry 1 *vs.* Table 2, entry 1). Significant rate enhancement was also seen for the polymerizations of *n*BA, *t*BA, DMA and AMO, and a high degree of controllability remained (Table 1, entries 3–15 *vs.* Table 2, entries 2–6). More importantly, the (salen)Co–CO_2_CH_3_ complex not only could mediate the polymerization of conjugated acrylates and acrylamides under visible light irradiation but also was suitable for the polymerization of typical non-conjugated vinyl acetate (VAc). The bulk polymerization of VAc gave PVAc of narrow polydispersity with excellent control over molecular weight (Table 2, entry 7). However, the polymerization was a little bit slow and a gel was formed after 39% conversion due to high viscosity. To achieve high conversion and avoid gel formation, the load of VAc relative to **I** was reduced to 200 equivalents and polymerization was conducted in DMSO-*d*_6_. In this case, PVAc with 78% conversion was obtained with moderate control over the molecular weight and polydispersity (Table 2, entry 8). Thus, (salen)Co–CO_2_CH_3_ is an alternate versatile catalyst to control the polymerization of both “more active” monomers (acrylates and acrylamides) and “less activated” monomers (VAc) under visible light irradiation.

**Table 2:** Visible light-initiated polymerization with **I** in the presence of TPO at ambient temperature^*a*^

Entry	Monomer (equiv.)	*t* (h)	Conv.^*b*^ (%)	*M*_n,th_ ^*c*^	*M*_n,GPC_ ^*d*^	*M*_w_/*M*_n_ ^*d*^
1	MA (600)	8	76	39 900	37 200	1.25
2	*n*BA (600)	8	81	62 900	55 300	1.23
3	*t*BA (600)	5	80	62 200	59 300	1.24
4	DMA (600)	2	75	45 300	40 000	1.22
5	DEA (600)	3	71	55 000	47 200	1.30
6	AMO (200)	3	87	25 200	23 800	1.24
7^*e*^	VAc (600)	100	39	20 800	20 600	1.20
8^*f*^	VAc (200)	64	78	14 100	18 600	1.33

^*a*^A mixture of **I** (1 equiv.), TPO (1 equiv.) and monomer was irradiated with [M]_0_ = 1.0 M in benzene-*d*_6_. A 500 W Xe lamp was used as the light source with a 420–780 nm filter, and the light intensity was 3 mW cm^–2^.

^*b*^The monomer conversion was determined based on the ^1^H NMR spectra.

^*c*^ *M*_n,th_ = *M*_w(_ **_I_**_)_ + *M*_w(Monomer)_ × ratio × conv. (%), where ratio refers to the ratio of monomer to **I**.

^*d*^Determined using gel permeation chromatography in DMF, calibrated against poly(methyl methacrylate) standards.

^*e*^Polymerization in bulk.

^*f*^[M]_0_ = 2.0 M in dimethyl sulfoxide-*d*.

### Kinetics and possible mechanism

**Scheme 2:**
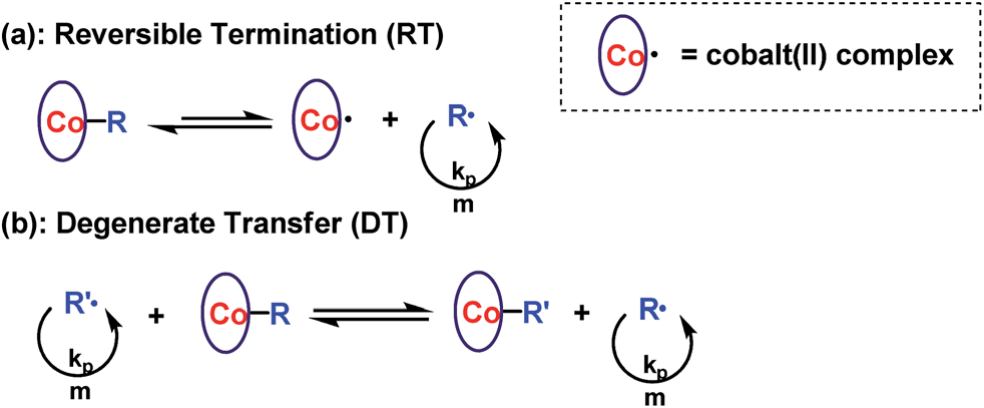
(a) Reversible termination and (b) degenerate transfer mechanism in cobalt-mediated radical polymerization.

Reversible termination (RT) and degenerate transfer (DT) are two competing mechanisms that occur in OMRP and cobalt-mediated radical polymerization (Scheme 2).^5,18b,24^ Dormant species (organocobalt complexes) are the exclusive source of radicals in RT, while the concentration of chain radicals in DT is mainly dependent on the concentration of an external radical source. The rate of polymerization *via* a DT process approaches the rate of a regular free radical polymerization, which is much faster than that in a RT process.^18*b*^ In order to investigate the mechanism, the kinetics of polymerization mediated by **I** under different conditions (irradiated by Xe lamp, CFL, or addition of TPO) were investigated (Fig. 3a). Mixing **I** with 1 equivalent of TPO in the photo-induced polymerization resulted in rapid first order kinetics with a conversion of 58% after only 4.5 h (Fig. 3a, blue dots). On the other hand, a much slower rate was obtained without addition of TPO (Fig. 3a, black and red dots). The radical concentration was calculated to be ∼8.8 × 10^–10^ M from the slope of 0.043 h^–1^ and *k*_p_ (MA at 298 K)^25^ of 13 500 M^–1^ S^–1^ when irradiated by the Xe lamp, which was similar to that using the CFL (1.0 × 10^–9^ M), and one order of magnitude smaller than that in the presence of TPO (6.2 × 10^–9^ M). The lower concentration of propagation chain radicals in the absence of TPO arose from the fact that photolysis of organocobalt salen was the sole pathway for the formation of chain radicals, and the resulting cobalt(ii) reversibly grabbed the chain radicals.

**Fig. 9:**
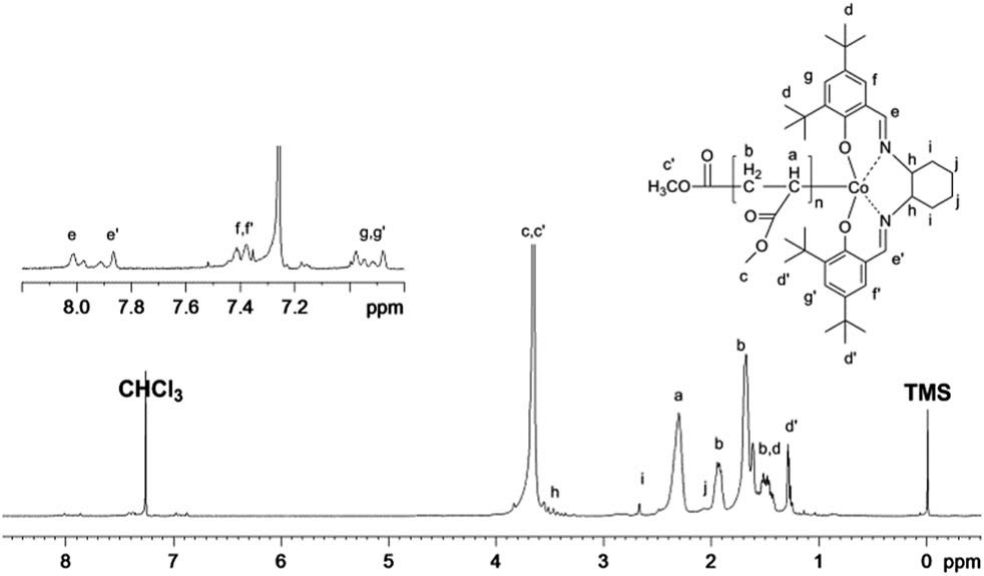
^1^H NMR spectrum of PMA synthesized by visible light-initiated polymerization with Xe lamp irradiation at ambient temperature. [MA]_0_ = 1.0 M, [MA]_0_/[(salen)Co–CO_2_CH_3_]_0_ = 200/1, conversion = 50%. *M*_n,GPC_ = 10 300, *M*_n,th_ = 9270, *M*_n,NMR_ = 9530, *M*_w_/*M*_n_ = 1.10. *M*_n,th_ = *M*_w(_ **_I_**_)_ + *M*_w(MA)_ × ratio × conv. (%); *M*_n,NMR_ = *I* ^2.3ppm^/(*I* ^7.9ppm^/2) × *M*_w(MA)_ + *M*_w(_ **_I_**_)_, where *I* ^2.3ppm^ and *I* ^7.9ppm^ are the integrals of the signals at *δ* 2.3 ppm and 7.9 ppm corresponding to the methane group of MA and the imine group of salen, respectively.

**Fig. 10:**
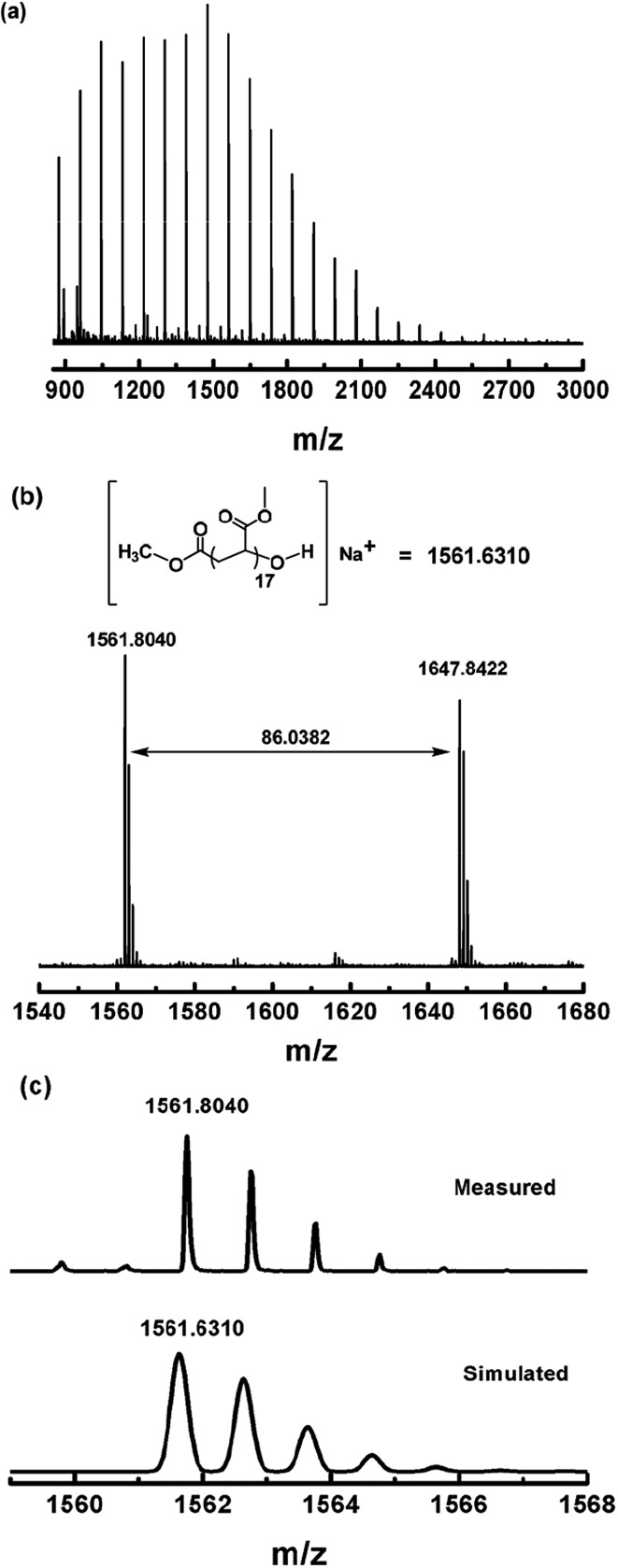
(a) Matrix-assisted laser desorption ionization time-of-flight mass spectrometry (MALDI-TOF-MS) of PMA after modification of the ω polymer end (*M*_n,GPC_ = 3450, *M*_w_/*M*_n_ = 1.16). (b) The expanded main peak series and theoretical molecular weight. (c) The simulation of the isotopic pattern.

To further understand the polymerization mechanism, the associative equilibrium constant for the formation of organocobalt complexes was evaluated ((salen)Co(ii) + R˙ ⇌ (salen)Co–R, *K*_eq_ = [(salen)Co–R]_eq_/([(salen)Co(ii)]_eq_ × [R˙])). [(salen)Co–R]_eq_ and [(salen)Co(ii)]_eq_ are the stationary state concentrations of dormant and deactivated species during the polymerization. A certain amount of (salen)Co(ii) (>5 mol% of the initial concentration of **I**) was added at the beginning of the polymerization in order to shift the activation–deactivation equilibrium towards the dormant species, so that only a tiny proportion of (salen)Co–CO_2_CH_3_ was dissociated during the polymerization. Thus, the equilibrium concentration of [(salen)Co–R]_eq_ and [(salen)Co(ii)]_eq_ was assumed to be equal to the initial concentration of [(salen)Co–CO_2_CH_3_]_0_ and [(salen)Co(ii)]_0_. To determine the *K*_eq_, 8% and 16% excesses of (salen)Co(ii) to (salen)Co–CO_2_CH_3_ were added before the irradiation, and the polymerization kinetics are shown in Fig. 4. Both the polymerizations gave linear consumption of MA with a decreased polymerization rate due to the addition of excess (salen)Co(ii). The radical concentrations were calculated to be 3.56 × 10^–10^ M and 1.83 × 10^–10^ M, respectively. The ratio of [(salen)Co–CO_2_CH_3_]_0_/[(salen)Co(ii)]_0_ combined with the radical concentration gave the equilibrium constants *K*_eq_ = 3.5 × 10^10^ M^–1^ and 3.4 × 10^10^ M^–1^, respectively. The *K*_eq_ was three orders of magnitude larger than that measured for the thermal polymerization of MA at 60 °C mediated by (salen)Co(ii) (*K*_eq_ ∼ 2.4 × 10^7^ M^–1^),^17*b*^ and close to the value for thermal polymerization by the cobalt porphyrin system (*K*_eq_ ∼ 8.7 × 10^9^ M^–1^).^26^ With such a large *K*_eq_, when TPO was added, the major radical source would mainly be external TPO rather than organocobalt (salen)Co–CO_2_CH_3_. Thus, the visible light-initiated polymerization mediated by complex **I** without TPO is suggested to mainly undergo a RT process, while a DT process is predominant in photopolymerization in the presence of TPO. Furthermore, the addition of TPO had a negligible influence on α end fidelity because only a tiny amount of polymer chain derived from the TPO segment was found in the MALDI-TOF-MS detection (ESI, Fig. 9S & 10S†).

**Fig. 11:**
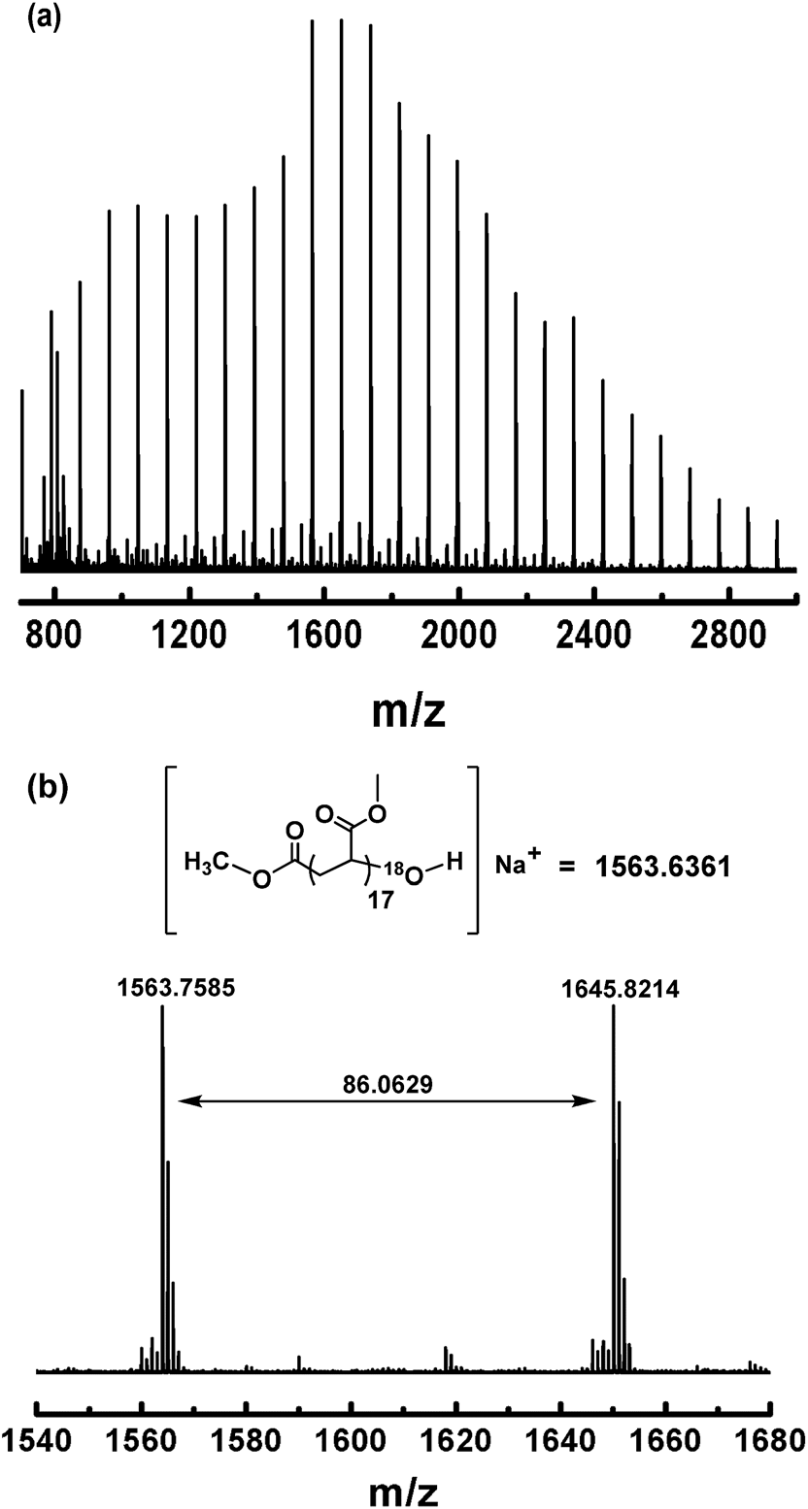
(a) Matrix-assisted laser desorption ionization time-of-flight mass spectrometry (MALDI-TOF-MS) of PMA (*M*_n,GPC_ = 3870, *M*_w_/*M*_n_ = 1.19) after modification of the ω polymer end using ^18^O_2_. (b) The expanded main peak series and theoretical molecular weight.

The living character of the visible light-initiated radical polymerization of MA was further demonstrated by the evolution of molecular weight *versus* conversion under different conditions (Fig. 3b). Gel permeation chromatography analysis revealed a linear increase in molecular weight with respect to conversion of MA under all conditions. The polydispersity index was small (*M*_w_/*M*_n_ < 1.25) from the early stages of polymerization up to high MA conversions in all cases. The GPC traces for photopolymerization under these conditions were all monomodal and symmetrical throughout the whole process (ESI, Fig. 11S–13S†). Furthermore, it is worth mentioning that in the thermal polymerization of MA mediated by (salen)Co(ii), there was a significant deviation between measured and calculated molecular weights, because the relatively small *K*_eq_ (2.4 × 10^7^ M^–1^) under thermal conditions resulted in incomplete conversion of cobalt(ii) complexes to organocobalt species.^17*b*^ However, the large *K*_eq_ of this photosystem at room temperature maintained nearly all the cobalt species as organocobalt complexes and fulfilled the ideal assumption of one polymer chain per cobalt. Therefore, the experimental molecular weights are all close to theoretical values, indicating high initiation efficiency for this visible light-initiated polymerization.

### Effect of visible light

Surprisingly, we found that after the initiation stage, the rate of polymerization was approximately the same with/without continuing irradiation. For example, after passing the initiation stage, one sample was protected from light by covering with aluminum foil and the other was continually irradiated under a light intensity of 3 mW cm^–2^, however, both of these two samples of DMA gave similar conversions (45% and 46%).

This observation definitely indicates that polymerization also occurs at a similar rate in the dark after initiation. This is interesting since visible light is essential for successful polymerization (Table 1, entry 8).

To assess the influence of visible light on the polymerization process at ambient temperature, MA was chosen as a model monomer and kinetic studies were conducted under three different sets of conditions. The molar ratio of MA to **I** was set at 200/1 each time, one sample was reacted under continuous Xe lamp irradiation and the second sample was subjected to a periodic light on–off process every 12 hours. The third sample was irradiated for the first 12 hours and then stored in the dark. The polymerization rate under continuous irradiation was slightly faster than that in the dark, and no significant rate difference was observed under continuous and periodic irradiation within 40 h (Fig. 5). So visible light of 3 mW cm^–2^ has a negligible influence over the polymerization rate after initiation. However, after a fairly long reaction time (more than 40 h), the polymerization under visible light irradiation showed a little bit higher monomer conversion than that in the dark (Fig. 5 & ESI Fig. 14S†). These results indicate that the Co–C bonds in (salen)Co–PMA and (salen)Co–PDMA could undergo homolysis to give propagating radicals even in the dark at ambient temperature, although **I** did not initiate polymerization in the dark (Table 1, entries 7–8). The bond dissociation energy of the Co–C bonds in Co–C(O)OR was much stronger than in Co–CHR_1_R_2_ due to the sp^2^-hybridized carbon in **I**.

### Polymer structure analysis

The chain growth process occurs through monomer insertion into the cobalt–carbon bond in the dormant organocobalt species. Thus, in the final polymer structure, the α ends were anticipated to be CO_2_CH_3_ from the (salen)Co–CO_2_CH_3_, while the ω ends should be Co(salen). In order to investigate the α ends of the polymer chains, a both deuterated and ^13^C-labeled organocobalt complex, (salen)Co–^13^CO_2_CD_3_, was synthesized under similar conditions (ESI†). It was characterized by ^1^H NMR, ^13^C NMR, ^2^D NMR and ESI-MS. The ^2^D NMR spectrum gave a clear peak at –1.315 ppm, corresponding to the deuterium atoms of the methyl group in (salen)Co–^13^CO_2_CD_3_ (Fig. 6a). After polymerization of MA, the isotopic-labeled methyl group in PMA shifted to –1.455 ppm, corresponding to the deuterium atoms at the α ends. The structure of PMA synthesized from (salen)Co–^13^CO_2_CD_3_ was further investigated by ^13^C NMR and compared with PMA prepared from regular (salen)Co–CO_2_CH_3_ (Fig. 7). The PMAs formed were highly linear, as no signal was detected in the ranges of *δ*(^13^C) = 37–40 and 47–49 ppm that are characteristic of acrylate branching.^27^ The two spectra were nearly identical except for the signal at 172 ppm, which was assigned to the ^13^C-labeled carbon atom in the ester group from the starting (salen)Co–^13^CO_2_CD_3_. Both the ^2^D NMR and ^13^C NMR detections demonstrated that the α ends of the formed polymers mediated by organocobalt salen complexes were CO_2_CH_3_ from the (salen)Co–CO_2_CH_3_.^13*b*^

To analyze the structure of the ω chain ends, GPC traces of PMA were measured by both refractive index and UV-visible detectors (Fig. 8 & ESI, Fig. 15S†). Polymers with the (salen)Co chromophore showed a strong UV-visible absorption band around 360 nm. The GPC traces of typical PMA samples measured by RID and UV-vis detectors were nearly the same, except for a delay in the RID signal due to the series connection of the equipment. The elution curves were narrow and symmetrical, indicating that each polymer chain contained one cobalt complex at the ω end.

The PMA obtained in our system was further characterized by ^1^H NMR (Fig. 9). The chemical shifts at 6.9 ppm, 7.4 ppm, and 7.8–8.0 ppm clearly demonstrate the presence of the (salen)Co group in the polymer chain. Furthermore, the molecular weight calculated from the ^1^H NMR analysis was 9530 g mol^–1^, which fits very well with the theoretical value (9270 g mol^–1^) and experimental molar mass (10 300 g mol^–1^), indicating high initiator efficiency.

### Polymer end group functionalization

**Scheme 3:**
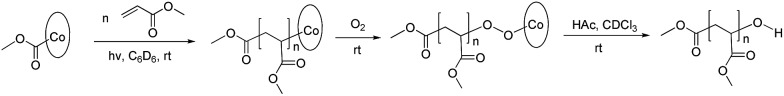
The strategy of modification of the ω end of the (salen)Co–PMA synthesized.

Photo-induced dioxygen insertion into a Co–C bond has been reported previously^28^ and Co(iii)–alkylperoxo complexes are important intermediates in cobalt-catalyzed hydrocarbon oxidation reactions.^29^ Our strategy for the modification of the ω end of the polymers obtained is illustrated in Scheme 3. As investigated in a periodic irradiation experiment, the Co–C bond in (salen)Co–PMA can undergo homolysis to give PMA˙ and (salen)Co(ii). Schiff-base cobalt(ii) complexes are well-known to bind dioxygen reversibly.^30^ Thus the photolysis of (salen)Co–PMA in the presence of dioxygen resulted in the formation of a Co(iii)–alkylperoxo complex, [(salen)Co–O–O–PMA], accompanied by a color change from yellow to dark brown (ESI, Fig. 16S†). The coordination of alkylperoxide to cobalt was presumed to weaken the O–O bond and assist with O–O bond cleavage.^29*b*^ Subsequently, hydrolysis of the (salen)Co–O–O–PMA complex under acidic conditions led to the formation of PMA with a terminal hydroxyl group (Scheme 3). Fig. 10a shows the MALDI-TOF-MS spectrum of the OH group-functionalized PMA sample (*M*_n,GPC_ = 3450, *M*_w_/*M*_n_ = 1.16) with a series of molecular ion peaks, regularly separated by the molar mass of the MA monomer. Experimental isotopic mass values of the main peak series were equal to those expected for the PMA with CH_3_CO_2_^–^ at the α end and a hydroxyl group at the ω end, plus a sodium ion from externally-added salt for ionization, as shown in the upper part of Fig. 10b. More specifically, the observed isotopic pattern (*m*/*z*) of the main series at *m*/*z* = 1561.8040 g mol^–1^ indicates the assigned species with a DP (degree of polymerization) of 17, and matches well with the simulated pattern, *m*/*z* = 1561.6310 g mol^–1^ (Fig. 10c). The observed isotopic pattern also agrees well with the simulation result.

To gain more evidence for the modification process (Scheme 3), an isotopic labeling experiment was conducted by reaction of (salen)Co–PMA with ^18^O_2_. The resulting PMA–^18^OH was also characterized by MALDI-TOF-MS (Fig. 11). The end-capped sample also gave a series of molecular ion peaks separated by MA units. The absolute mass of each peak clearly indicates the ^18^O-labeled structure. Thus, the isotopic labeling and MALDI-TOF-MS analysis both suggest that the end group has been successfully functionalized by an OH group, which would be highly useful for further application.

### Synthesis of block copolymers

To further probe the living nature of this visible light-initiated system, as well as to provide additional evidence for the presence of cobalt salen complexes at the ω chain ends, block copolymers were prepared by sequential polymerization of acrylates and acrylamides. MA and DMA were chosen as model monomers. Three (salen)Co–PMAs with narrow molecular weight distributions were prepared by photopolymerization under different conditions (irradiated by Xe lamp or CFL, or with addition of TPO) and used as macroinitiators (ESI, Fig. 17S–19S†). After addition of fresh DMA, all the polymerizations under these conditions proved to be well-behaved processes leading to the desired PMA-*b*-PDMA with predetermined molar masses and low polydispersities (Fig. 20S†). The GPC traces all showed substantial shift with negligible residual macroinitiators after block copolymerization. The successful block copolymer synthesis further indicated efficient reversible activation of the cobalt–carbon chain end and minimal termination under all polymerization conditions.

## Conclusions

In summary, we have demonstrated that (salen)Co–CO_2_CH_3_ acts as a versatile photoinitiator to mediate the polymerization of acrylates, acrylamides and non-conjugated VAc. All the polymerizations showed controlled behavior, evidenced by the formation of polymers with predetermined molecular weights and low polydispersities. The equilibrium constant ((salen)Co(ii) + R˙ ⇌ (salen)Co–R, *K*_eq_ = [(salen)Co–R]_eq_/([(salen)Co(ii)]_eq_ × [R˙])) between the organo radical, cobalt(ii) and the organocobalt(iii) species was determined to be 3.5 × 10^10^ M^–1^. Addition of 1 equivalent of TPO dramatically increased the polymerization rate while maintaining high controllability, indicating a switch from RT polymerization to a DT mechanism. Visible light was found to be essential for the initiation but showed a negligible effect during propagation, which might result from the difference in the cobalt–carbon bond energy in (salen)Co–C(sp^2^) and (salen)Co–C(sp^3^). Polymer structure analysis demonstrated the presence of (salen)Co segments in the polymer chain ω end and –CO_2_CH_3_ segments in the polymer chain α end. Efficient block copolymer synthesis under Xe lamp, CFL, and with addition of TPO, further confirmed the versatile capability of this novel complex in controlled/living radical polymerization. The convenient synthesis of salen complexes offers a unique and appreciable approach for the preparation of functional polymeric materials.

## References

[cit1] Hawker C. J., Bosman A. W., Harth E. (2001). Chem. Rev..

[cit2] Wang J. S., Matyjaszewski K. (1995). J. Am. Chem. Soc..

[cit3] Moad G., Rizzardo E., Thang S. H. (2008). Polymer.

[cit4] David G., Boyer C., Tonnar J., Ameduri B., Lacroix-Desmazes P., Boutevin B. (2006). Chem. Rev..

[cit5] Debuigne A., Poli R., Jerome C., Jerome R., Detrembleur C. (2009). Prog. Polym. Sci..

[cit6] Leibfarth F. A., Mattson K. M., Fors B. P., Collins H. A., Hawker C. J. (2013). Angew. Chem., Int. Ed..

[cit7] Magenau A. J. D., Strandwitz N. C., Gennaro A., Matyjaszewski K. (2011). Science.

[cit8] Tasdelen M. A., Uygun M., Yagci Y. (2011). Macromol. Rapid Commun..

[cit9] Quinn J. F., Barner L., Barner-Kowollik C., Rizzardo E., Davis T. P. (2002). Macromolecules.

[cit10] Guillaneuf Y., Bertin D., Gigmes D., Versace D.-L., Lalevée J., Fouassier J.-P. (2010). Macromolecules.

[cit11] Yan J. F., Li B., Zhou F., Liu W. M. (2013). ACS Macro Lett..

[cit12] Benaglia M., Chiefari J., Chong Y. K., Moad G., Rizzardo E., Thang S. H. (2009). J. Am. Chem. Soc..

[cit13] Zhao Y. G., Yu M. M., Fu X. F. (2013). Chem. Commun..

[cit14] Chiang L., Allan L. E. N., Alcantara J., Wang M. C. P., Storr T., Shaver M. P. (2014). Dalton Trans..

[cit15] Tokunaga M. (1997). Science.

[cit16] Qin Z. Q., Thomas C. M., Lee S., Coates G. W. (2003). Angew. Chem., Int. Ed..

[cit17] Sherwood R. K., Kent C. L., Patrick B. O., McNeil W. S. (2010). Chem. Commun..

[cit18] Lu Z., Fryd M., Wayland B. B. (2004). Macromolecules.

[cit19] Leung W. H., Chan E. Y. Y., Chow E. K. F., Williams I. D., Peng S. M. (1996). J. Chem. Soc., Dalton Trans..

[cit20] Marzilli L. G., Summers M. F., Brescianipahor N., Zangrando E., Charland J. P., Randaccio L. (1985). J. Am. Chem. Soc..

[cit21] Coveney D. J., Patel V. F., Pattenden G., Thompson D. M. (1990). J. Chem. Soc., Perkin Trans. 1.

[cit22] Jiang W. D., Lu L. C., Cai Y. L. (2007). Macromol. Rapid Commun..

[cit23] Goto A., Kwak Y., Fukuda T., Yamago S., Iida K., Nakajima M., Yoshida J. (2003). J. Am. Chem. Soc..

[cit24] Debuigne A., Champouret Y., Jerome R., Poli R., Detrembleur C. (2008). Chem.–Eur. J..

[cit25] Barner-Kowollik C., Beuermann S., Buback M., Castignolles P., Charleux B., Coote M. L., Hutchinson R. A., Junkers T., Lacik I., Russell G. T., Stach M., van Herk A. M. (2014). Polym. Chem..

[cit26] Peng C. H., Li S., Wayland B. B. (2009). Inorg. Chem..

[cit27] Ahmad N. M., Heatley F., Lovell P. A. (1998). Macromolecules.

[cit28] Kendrick M. J., Alakhdar W. (1987). Inorg. Chem..

[cit29] Chavez F. A., Rowland J. M., Olmstead M. M., Mascharak P. K. (1998). J. Am. Chem. Soc..

[cit30] Huber A., Muller L., Elias H., Klement R., Valko M. (2005). Eur. J. Inorg. Chem..

