# Supplementary material for: A well-defined, versatile photoinitiator (salen)Co–CO2CH3 for visible light-initiated living/controlled radical polymerization
Source: Chem Sci. 2015 Mar 5;6(5):2979–88. doi: 10.1039/c5sc00477b (PMC5656039; doi:10.1039/c5sc00477b)
Supplement: Supplementary file 1 [file SC-006-C5SC00477B-s001.pdf]

Electronic Supplementary Material (ESI) for Chemical Science.
This journal is © The Royal Society of Chemistry 2015

A Well-defined Versatile Photoinitiator (salen)Co-CO_2CH_3 for Visible Light Initiated Living/Controlled Radical Polymerization

Yaguang Zhao,† Mengmeng Yu,† Shuailin Zhang,† Zhenqiang Wu,† Yuchu Liu,† Chi-How Peng,*‡ and Xuefeng Fu*†

†Beijing National Laboratory for Molecular Sciences, State Key Lab of Rare Earth Materials Chemistry and Applications, College of Chemistry and Molecular Engineering, Peking University, Beijing, 100871, China

‡Department of Chemistry and Frontier Research Center on Fundamental and Applied Sciences of Matters, National Tsing-Hua University, Hsinchu, 30013, Taiwan

Experimental

Materials.

Methyl acrylate (MA, Alfa Aesar, 99%), n-butyl acrylate (nBA, Alfa Aesar, 98+%), and tert-butyl acrylate (tBA, Alfa Aesar, 99%) were purified by passing through a neutral alumina column and distilled under reduced pressure to remove the inhibitor. *N,N*-dimethylacrylamide (DMA, Alfa, 99.5%), *N,N*-diethylacrylamide (DEA, TCI, >98.0%), and *N*-acryloylmorpholine (AMO, TCI, >98.0%) were distilled under reduced pressure and stored in the refrigerator before use. Cobalt(II)[N,N'-bis(3,5-di-tert-butylsalicylidene)-1,2-cyclohexanediamine] ((salen)Co(II), Aldrich, >99%), (2,4,6-trimethylbenzoyl)diphenylphosphine oxide (TPO, TCI, 98.0%), Potassium peroxymonosulfate (Oxone®, Alfa), Methanol (J&K, 99.9%), toluene (Sinopharm Chemical Reagent Co., Ltd, 99.8%) and $^{18}O_2$ (Beijing Gaisi Chemical Gases Center, 97%) were used as received.

Light Source.

A 500 W xenon lamp (CEL-S500, Aulight, Beijing, China) was used as the light source with a 420−780 nm filter to give visible light. The intensity of the irradiation is modulated by employing neutral density filters with different transmittance. The intensity of visible light irradiation was measured by a FZ-A radiometer (Photoelectric Instrument Factory of Beijing Normal University) equipped with a 400−1000 nm sensor.

A household CFL (compact fluorescent lamp, 27 W) was used as the light source; the sample was placed at an approximate distance of 5 cm to the lamp, and the light intensity was measured to be 3−5 mW/cm^2.

Sunlight was directly used as light source without any optical filters. The intensity of solar irradiation varied between 0.8 and 10 mW/cm^2 within the wavelength range of 400-1000 nm.

Synthesis of complex (salen)Co-CO_2CH_3 I and (salen)Co-$^{13}CO_2CD_3$.

Methanol (300 μL) was added into 8.5 mL of a toluene solution containing (salen)Co(II) (0.09 mmol, 55 mg), and Oxone® (0.44 mmol, 135mg). The mixture was reacted for 0.5 h at room temperature. Subsequently, the mixture was filtered, added with $Na_3PO_4 \cdot 12H_2O$ (1.71 mmol, 650mg), degassed by three freeze-pump-thaw cycles and refilled with carbon monoxide (CO, 1 atm). The sample was shielded

from light and left stirring for 10 minutes at room temperature. After the solvent was removed under reduced pressure, the crude product was purified by column chromatography (basic alumina, CH_2Cl_2 as eluent). (salen)Co-CO_2CH_3 **I** (24 mg, 40% yield) was obtained as dark green solid. Recrystallization using CH_2Cl_2/hexane gave dark green crystal suitable for single-crystal X-ray diffraction analysis. (salen)Co-CO_2CH_3 ^{1}H NMR ($CDCl_3$, 500 MHz, 303K, δ): 8.06 (s, 1H), 7.86 (s, 1H), 7.40 (t, ^4J = 2.5Hz, 2H), 7.02 (d, ^4J = 2.5 Hz, 1H), 6.98 (d, ^4J = 2.5 Hz, 1H), 3.68 (s, 3H), 3.44 (m, 2H), 2.76 (m, , 2H), 2.04 (m, 2H), 1.65 (m, 2H), 1.58 (s, 9H), 1.54 (s, 9H), 1.46 (m, 2H), 1.31 (s, 9H), 1.30 (s, 9H). ^{1}HNMR (C_6D_6, 400 MHz, 303K, δ): 7.78 (m, 2H), 7.63 (s, 1H), 7.46 (s, 1H), 7.18 (t, J = 2.5 Hz, 2H), 3.44 (s, 3H), 3.12 (m, 1H), 2.27 (m, 1H), 2.04 (s, 9H), 2.00 (s, 9H), 1.72 (m, 2H), 1.41 (s, 9H), 1.40 (s, 9H), 1.34 (m, 4H). ^{13}C NMR ($CDCl_3$, 125 MHz, 303K, δ): 24.76, 24.87, 29.42, 29.59, 30.02, 31.52, 34.10, 34.13, 36.22, 56.21, 69.26, 73.07, 118.83, 119.98, 127.28, 127.37, 129.12, 136.42, 136.83, 141.92, 142.45, 161.29, 161.47, 164.02, 164.31. ESI-HRMS calcd for $C_{38}H_{55}CoN_2O_4$ $[M]^+$: 662.34883, found: 662.35060.

(salen)Co-$^{13}CO_2CD_3$ was synthesized under similar conditions simply by replacing CH_3OH with CD_3OD and CO with ^{13}CO. ^{1}H NMR ($CDCl_3$, 500 MHz, 303K, δ): 8.06 (s, 1H), 7.86 (s, 1H), 7.40 (t, ^4J = 2.5Hz, 2H), 7.02 (d, ^4J = 2.5 Hz, 1H), 6.98 (d, ^4J = 2.5 Hz, 1H), 3.44 (m, 2H), 2.76 (m, , 2H), 2.04 (m, 2H), 1.65 (m, 2H), 1.58 (s, 9H), 1.54 (s, 9H), 1.46 (m, 2H), 1.31 (s, 9H), 1.30 (s, 9H). ^{13}C NMR ($CDCl_3$, 125 MHz, 303K, δ): 24.76, 24.87, 29.42, 29.59, 30.02, 31.52, 34.10, 34.13, 36.22, 69.26, 73.07, 118.83, 119.98, 127.28, 127.37, 129.12, 136.42, 136.83, 141.92, 142.45, 161.29, 161.47, 162.73, 164.02, 164.31. ESI-HRMS calcd for $C_{37}H_{52}CoN_2O_4{}^{13}CD_3$ $[M]^+$: 666.37102, found: 666.36959.

Table 1S. Crystal Data of (salen)Co-CO_2CH_3 **I**:

Compound reference	(salen)Co-CO_2CH_3
Chemical formula	$C_{38}H_{55}CoN_2O_4$
Formula Mass	662.77
Crystal system	Monoclinic
a/Å	17.569(4)
b/Å	10.302(2)
c/Å	19.781(4)
α/°	90
β/°	100.629(2)
γ/°	90
Unit cell volume/ Å ^{3}	3518.8(12)
Temperature/K	173.1500
Space group	P121/n1
No. of formula units per unit cell, Z	4
Radiation type	MoKα
Absorption coefficient, μ/mm^{-1}	0.529
No. of reflections measured	23869

No. of independent reflections	8036
R_{int}	0.0422
Final R_1 values (($I > 2\sigma(I)$)	0.0698
Final $wR(F^2)$ values (($I > 2\sigma(I)$)	0.1519
Final R_1 values (all data)	0.0730
Final $wR(F^2)$ values (all data)	0.1534
Goodness of fit on F^2	1.260
CCDC number	1031130

Typical Procedure for Visible Light Initiated Polymerization.

A typical procedure for polymerization of acrylates, acrylamides and vinyl acetate mediated by organocobalt complex is as follows: (1) a certain amount of (salen)Co-CO_2CH_3 and monomers were mixed in C_6D_6 or DMSO-d_6 in a J. Young valve NMR tube; (2) after being thoroughly mixed and three freeze−pump−thaw cycles, the sample was refilled with nitrogen; (3) the sample was placed in a room temperature water bath and irradiated for a period of time. The progress of polymerization was followed by ^{1}H NMR measurement. When desired conversion (for the polymerization of DMA, DEA and AMO, benzaldehyde sealed in capillary tube was used as external reference) was reached, the reaction was stopped by exposure to air. The solvent and excess monomers were removed under vacuum. The resulting product without further purification was dissolved in DMF for GPC analysis.

Synthesis of Block Copolymers.

Block copolymers were synthesized by sequential polymerization of MA and DMA. PMA macroinitiators were synthesized by visible light initiated polymerization of MA mediated by (salen)Co-CO_2CH_3 under different conditions (irradiated by Xe lamp, CFL, or addition of TPO) at ambient temperature. After a desired conversion was obtained, the resulting PMA was used as macroinitiator for DMA under variable conditions. After desired conditions were reached, the polymerizations were stopped. Solvent and excess DMA was removed under vacuum. The resulting PMA-b-PDMA block copolymers were dissolved in DMF for GPC analysis without purification.

Characterization.

Conversions of monomers were determined by ^{1}H NMR spectrometry on a Bruker 400 MHz FT spectrometer in C_6D_6 or DMSO-d_6. ^{13}C NMR spectrum was recorded on a Bruker 500 MHz FT spectrometer. IR (film) was recorded with a Nicolet Avatar 330 FT-IR infrared spectrometer. The UV−vis spectra were acquired using a Shimadazu UV3100 spectrometer. Matrix-assisted laser desorption ionization time-of-flight mass spectroscopy (MALDI-TOF-MS) measurement was performed on a TOF/TOF 5800 system (AB SCIEX) with α-cyano-4-hydroxycinnamic acid (CHCA) as a matrix and sodium trifluoroacetate as the cationizing agent in positive reflection mode. Samples for the MALDI-TOF MS measurements were prepared by mixing the polymer (10 mM, 1μL), the matrix (48 mM,10μL) and the cationizing agent (10 mM, 10μL) in acetonitrile.

Gel permeation chromatography (GPC) was performed in an Agilent 1200 series system, equipped with two VARIAN PolarGel-M columns (300 × 7.5 mm), an Iso Pump (G1310A), a UV detector at 420 nm, and a differential refractive index detector (RID). The number-average molecular weight (M_n), weight-average molecular weight (M_w), and the polydispersity (PDI) were measured in DMF at 50 °C with a flow rate of 1.0 mL/min. A series of poly(methyl methacrylate)s (molecular weight range of 2710−679 000 g/mol, from Polymer Laboratories) were used as standards for calibration.

Characterization of (salen)Co-CO_2CH_3 I:

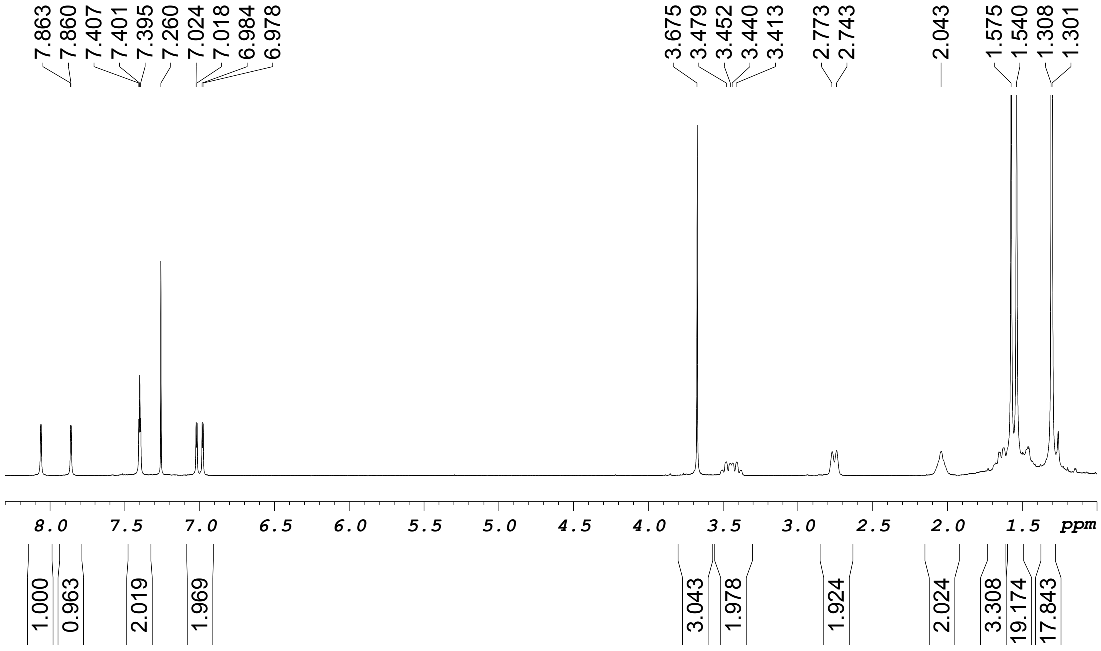

Figure 1S. ^{1}H NMR spectrum of (salen)Co-CO_2CH_3 in $CDCl_3$.

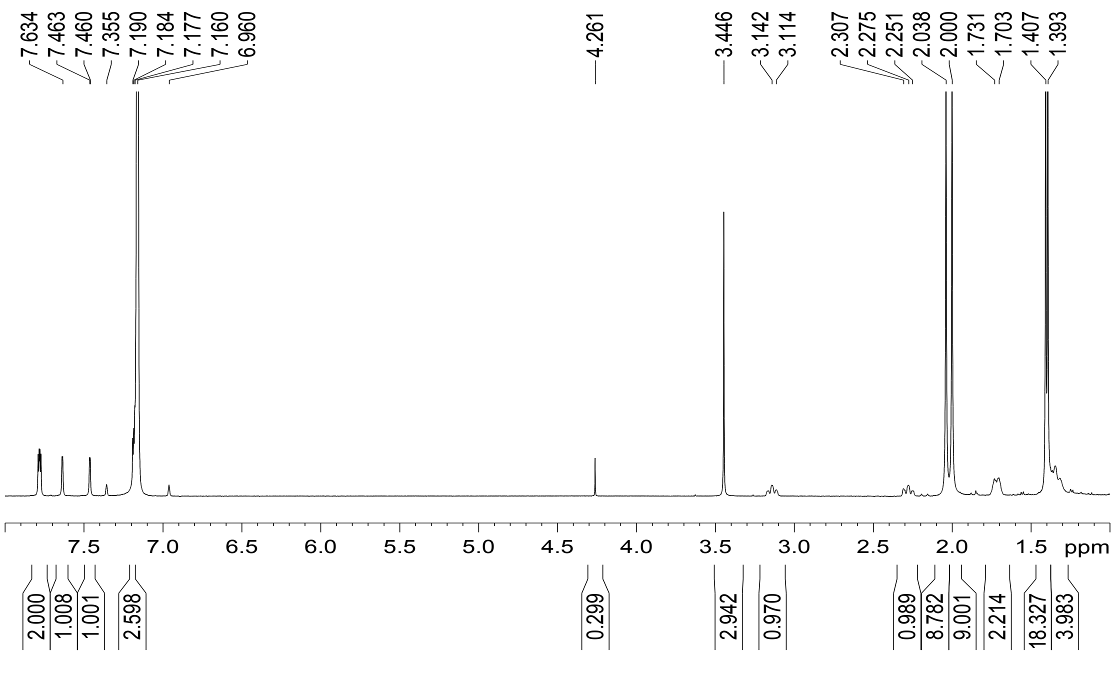

Figure 2S. ^{1}H NMR spectrum of (salen)Co-CO_2CH_3 in C_6D_6.

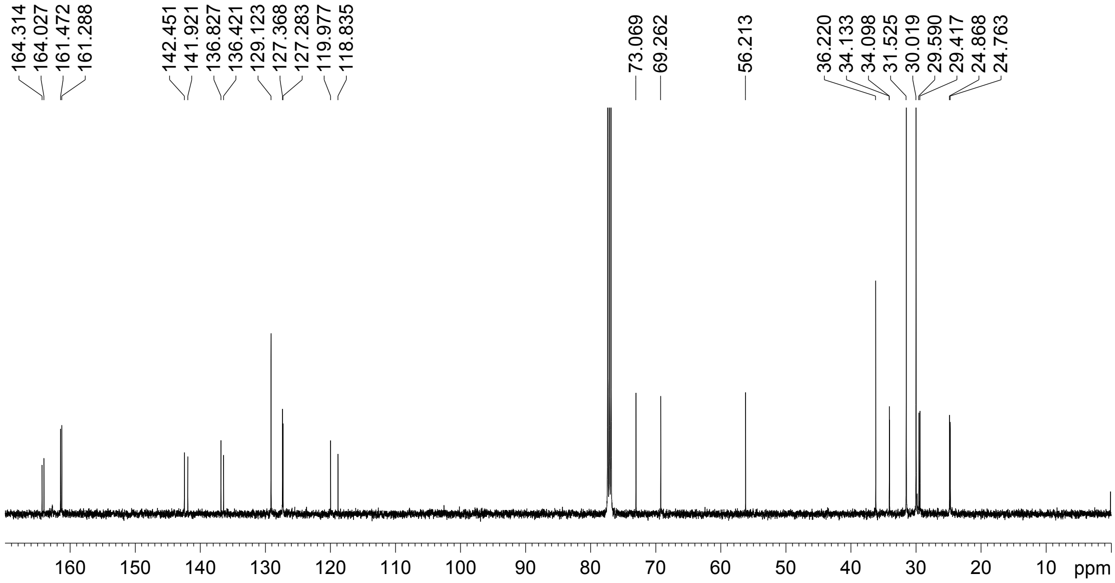

Figure 3S. ^{13}C NMR spectrum of (salen)Co-CO_2CH_3 in $CDCl_3$.

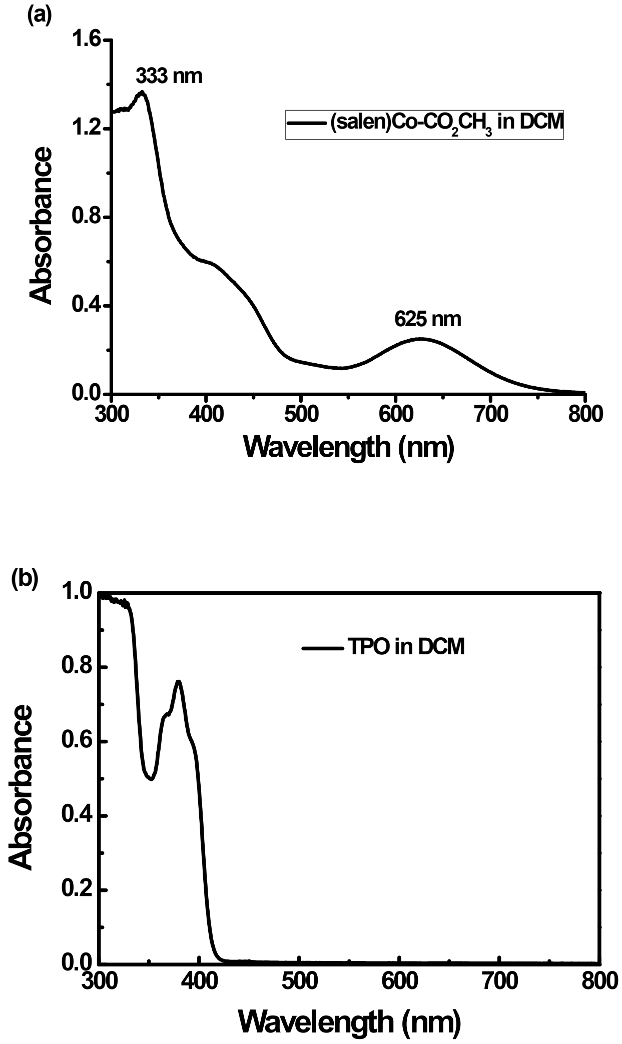

Figure 4S. UV-vis spectra of (a) (salen)Co-CO_2CH_3 **I** and (b) TPO in CH_2Cl_2.

Typical Colors of Different Compounds.

Both the polymerization of acrylates and transformation of **I** was followed by the color change. Here we show typical colors of different compounds in C_6D_6 during this research.

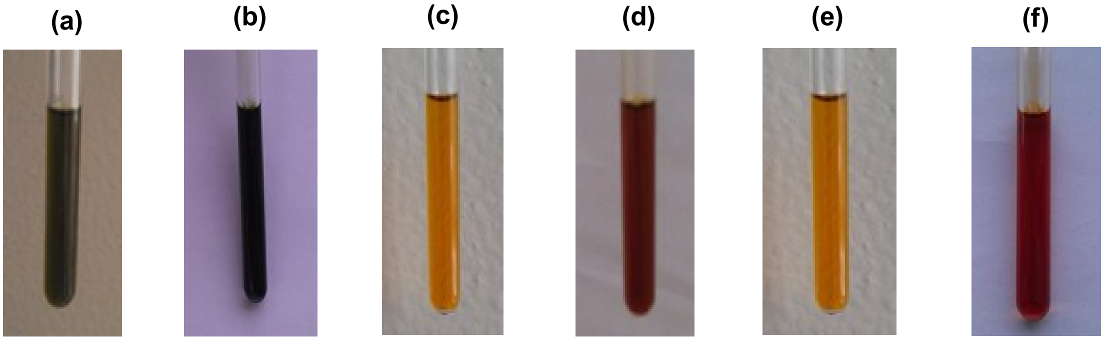

Figure 5S. Typical colors of several compounds during the research. (a) (salen)Co-CO_2CH_3, 1.6×10^{-3} M, light green; (b) (salen)Co-CO_2CH_3, 5×10^{-3} M, dark green; (c) (salen)Co-PMA, 1.6×10^{-3} M, yellow; (d) (salen)Co-PMA, 5×10^{-3} M, orange; (e) (salen)Co(II), 1.6×10^{-3} M, yellow; (e) (salen)Co(II), 5×10^{-3} M, red.

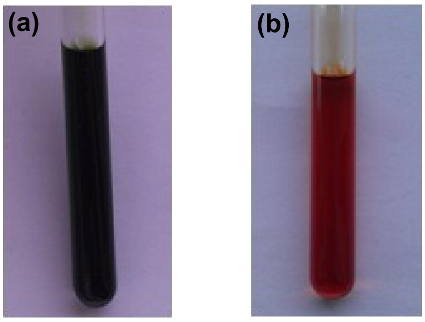

Figure 6S. Color change during radical trapping experiment: the benzene-d_6 solution of (salen)Co-CO_2CH_3 **I** (5 mM) and TEMPO (50 mM) was irradiated by visible light (I = 3 mW/cm^2 at 420−780 nm wavelength) at ambient temperature. (a) 0h; (b) 1h.

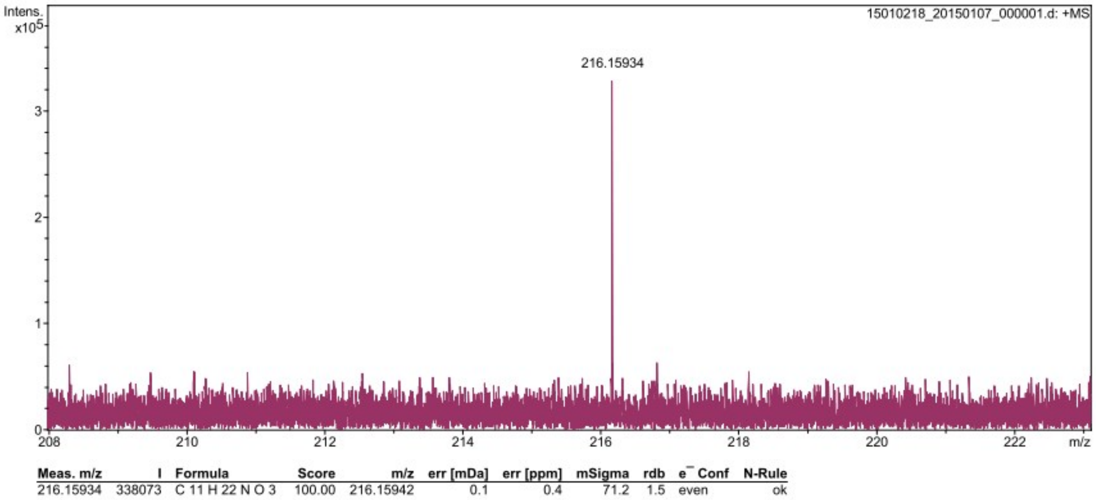

Figure 7S. ESI spectra of TEMPO-CO_2CH_3 formed according to procedure mentioned in photolysis of (salen)Co-CO_2CH_3 and TEMPO.

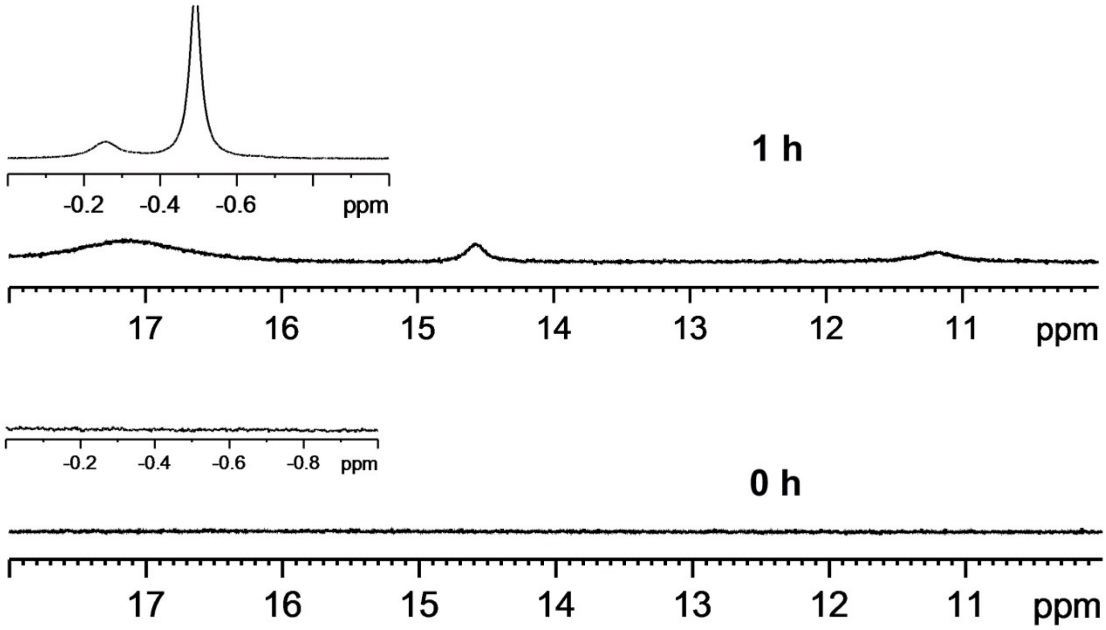

Figure 8S. ^{1}H NMR (400 MHz) spectra (low field) in C_6D_6 during the visible light irradiation of (TMP)Co-CO_2CH_3 in the presence of TEMPO indicating the formation of corresponding (salen)Co(II).

Influence of TPO to the α-end fidelity of final polymer

During the polymerization of acrylates under Xe lamp irradiation with addition of TPO, TPO was used to accelerate the polymerization while maintain good control over molecular weight and polydispersity. But since TPO could also initiate new polymer chain, it might influence the α-end fidelity by polymer chains with TPO sigments. To evaluate the α-end fidelity, two PMA samples were prepared by irradiation with addition of TPO and analyzed by MALDI-TOF-MS (Figure 9S & 10S). The results indicated that there were only one main series of peaks with MA

repeating unit. The experimental isotopic mass values of the main peak series agreed well with the theoretical values, as shown in the upper part of Figure 9S and 10S. The peak series referring to polymer chains initiated by TPO could be ignored as only tiny proportion of signal (< 5% of main peak series) was found. Thus when one equivalent of TPO was addition before polymerization, it could significantly increase the polymerization rate but gave insignificant influence to the α-end fidelity of final polymer.

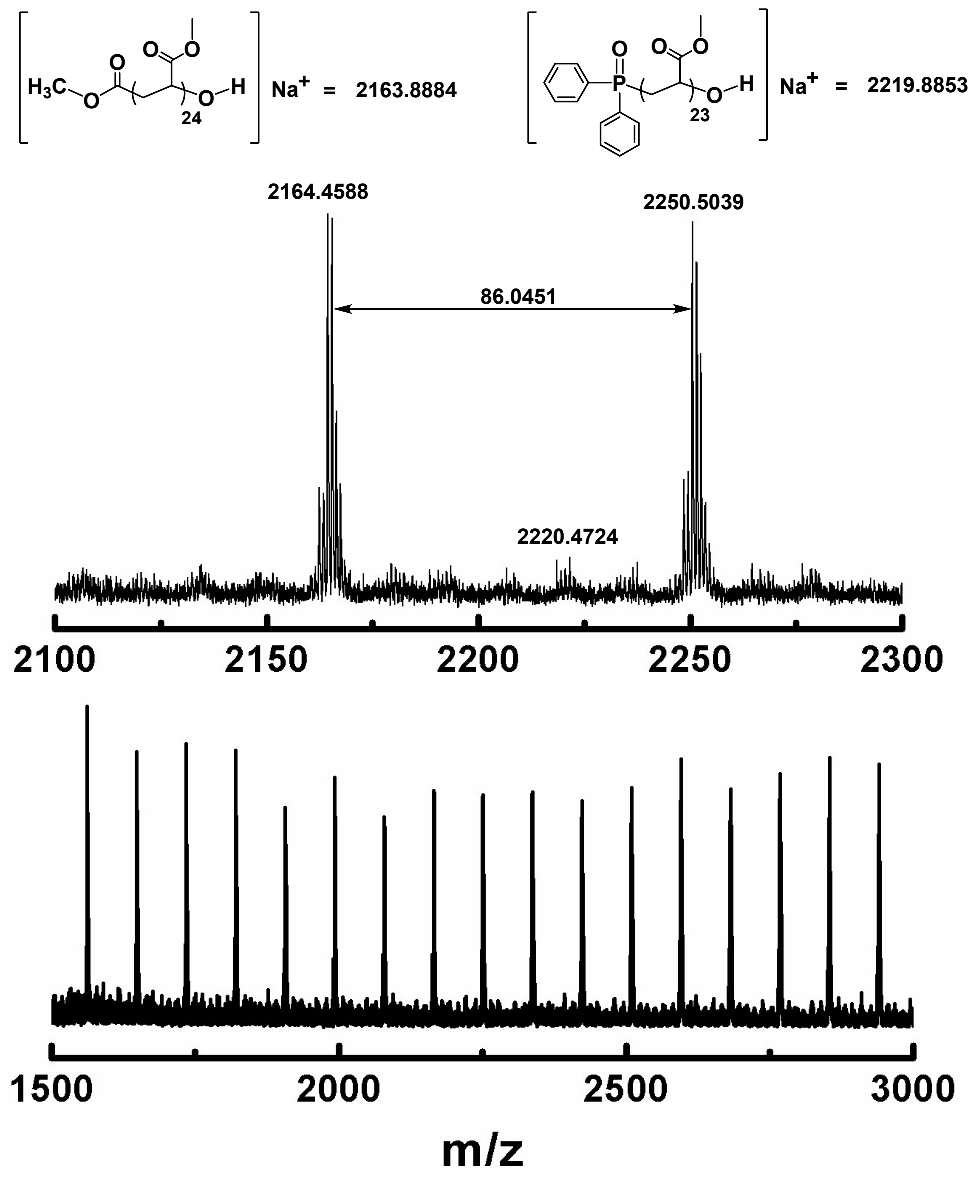

Figure 9S. Matrix-assisted laser desorption ionization time-of-flight mass spectroscopy (MALDI-TOF-MS) of PMA obtained from photo-LRP mediated by (salen)Co-CO_2CH_3 with addition of one equivalent TPO at room temperature. $[MA]_0$ = 1.0M, $[MA]_0/[Co\text{-}R]_0/[TPO]_0$ = 600/1/1, t = 1h, Conv% = 8.7%, $M_{n,th}$ = 5140, $M_{n,GPC}$ = 5630, M_w/M_n = 1.15.

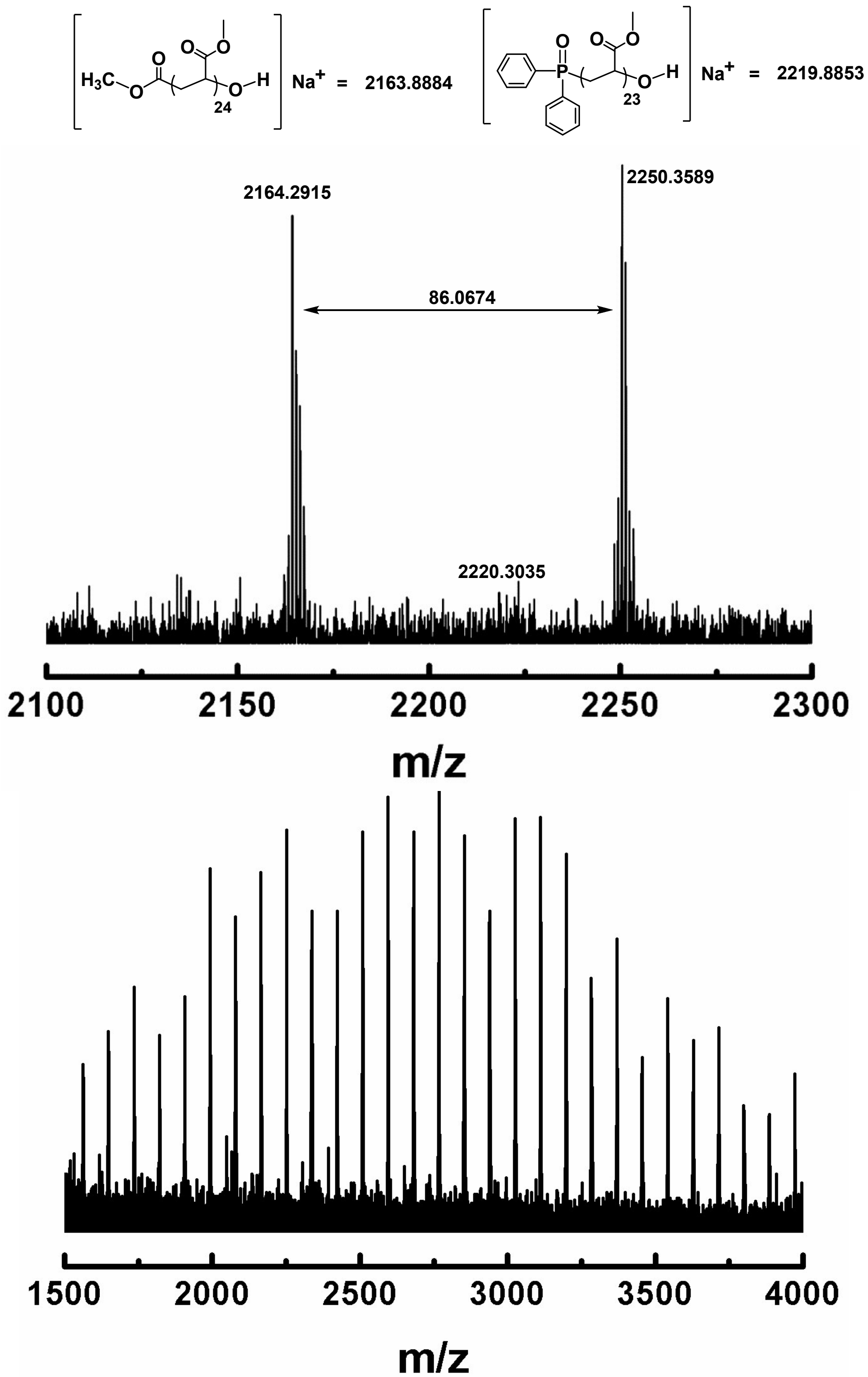

Figure 10S. Matrix-assisted laser desorption ionization time-of-flight mass spectroscopy (MALDI-TOF-MS) of PMA obtained from photo-LRP mediated by (salen)Co-CO_2CH_3 with addition of one equivalent TPO at room temperature. $[MA]_0$ = 1.0M, $[MA]_0/[Co\text{-}R]_0/[TPO]_0$ = 200/1/1, t = 4.5h, Conv% = 14.1%, $M_{n,th}$ = 3090, $M_{n,GPC}$ = 3430, M_w/M_n = 1.13.

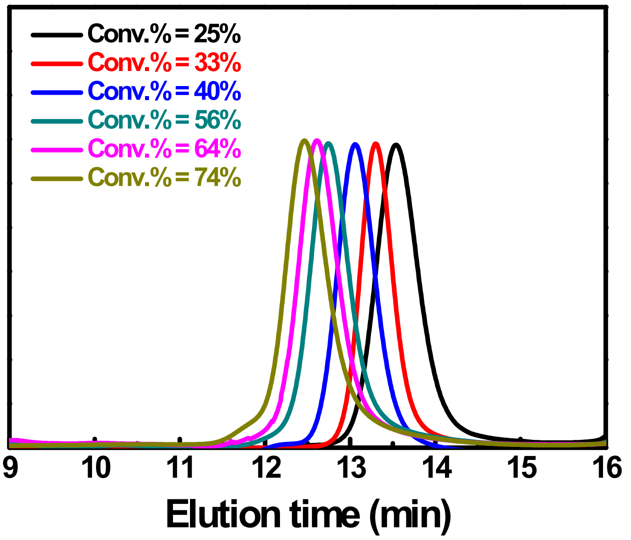

Figure 11S. GPC traces for photo-polymerization of MA in benzene with **I** under Xe lamp irradiation (3 mw/cm^2) at ambient temperature. Experimental conditions: $[MA]_0$ = 1.0 M; $[MA]_0/[\mathbf{I}]_0$ = 600/1.

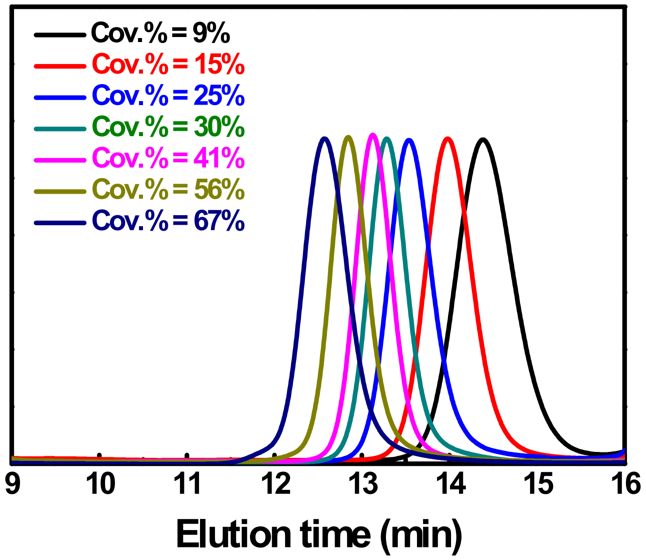

Figure 12S. GPC traces for photo-polymerization of MA in benzene with **I** under CFL irradiation (3-5 mw/cm^2) at ambient temperature. Experimental conditions: $[MA]_0$ = 1.0 M; $[MA]_0/[\mathbf{I}]_0$ = 600/1.

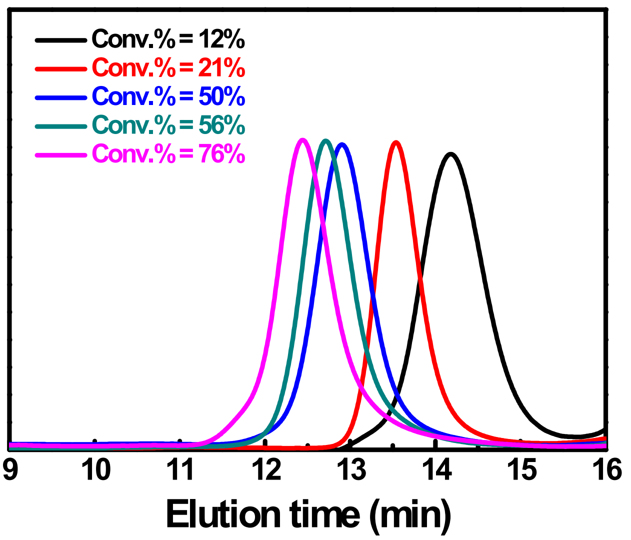

Figure 13S. GPC traces for photo-polymerization of MA in benzene with **I** under Xe lamp irradiation (3 mw/cm^2) with addition of 1 equivalent of TPO at ambient temperature. Experimental conditions: $[MA]_0 = 1.0$ M; $[MA]_0/[\mathbf{I}]_0/[TPO]_0 = 600/1/1$.

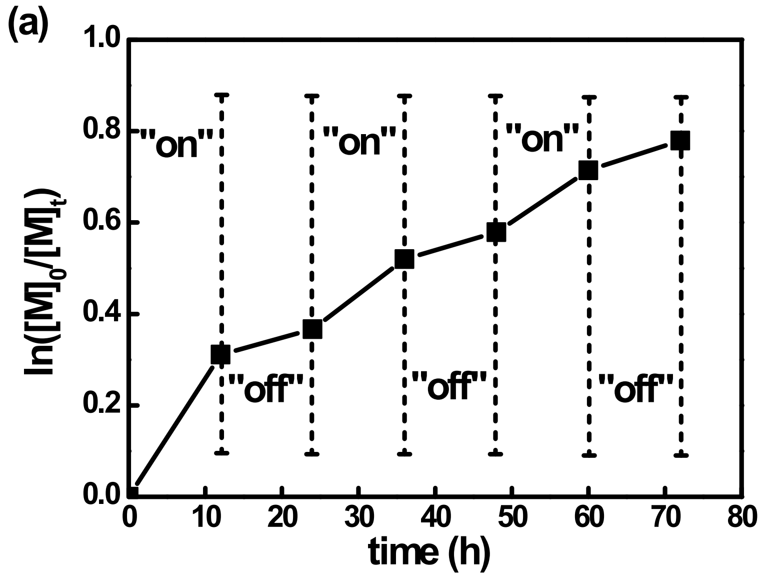

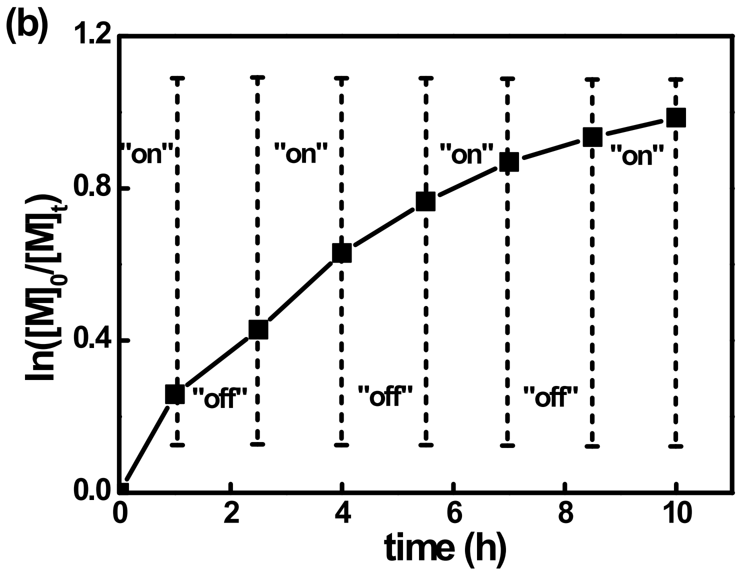

Figure 14S. Effect of visible light during the intermittent visible light initiated polymerization of MA and DMA in benzene mediated by **I** under Xe lamp irradiation (3 mw/cm^2) at ambient temperature: (a) $[MA]_0$ = 1.0 M; $[MA]_0/[\mathbf{I}]_0$ = 600/1; (b) $[DMA]_0$ = 1.0 M; $[DMA]_0/[\mathbf{I}]_0$ = 600/1.

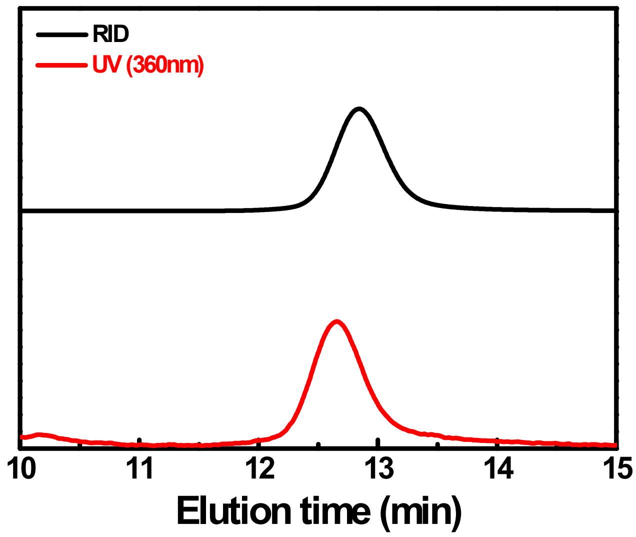

Figure 15S. Gel permeation chromatography (GPC) traces of the PMA produced by photo-CRP mediated by **I** ($M_{n,th}$ = 29600, $M_{n,GPC}$ = 28500, M_w/M_n = 1.06). Black line indicated the refractive index detection trace, and red line indicated the UV-visible (360 nm) detection trace.

Modification of ω end of (salen)Co-PMA.

After the visible light initiated polymerization of MA, excess MA and C_6D_6 was removed under vacuum. The resulting (salen)Co-PMA was dissolved in $CDCl_3$ in the

glove box and detected by ^1H NMR to ensure the complete removal of monomer. Then the solution was degassed by three freeze−pump−thaw cycles and back filled with $^{16}O_2$ or $^{18}O_2$ using vacuum line. After thoroughly mixed for 1 h, excess acetic acid was added. The solvent and acetic acid was removed under vacuum after 1h and the resulting product was analyzed by both MALDI-TOF-MS and GPC.

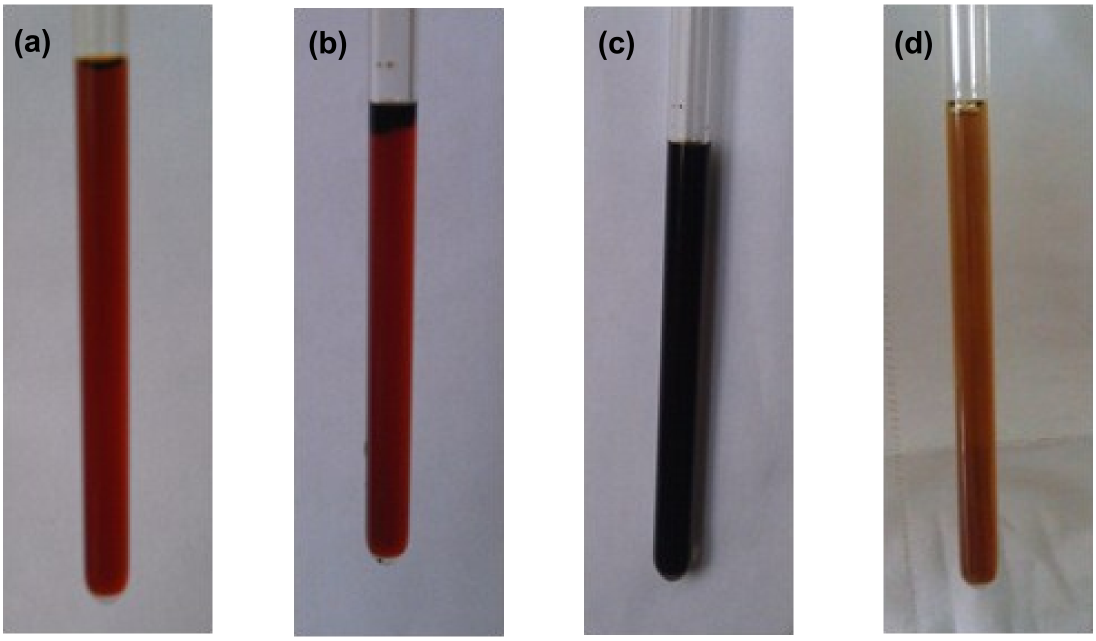

Figure 16S. Color change during the modification of ω end of (salen)Co-PMA : (a) before O_2 addition; (b) 0.5 min after O_2 addition; (c) 1 h after O_2 addition; (d) after acetic acid addition.

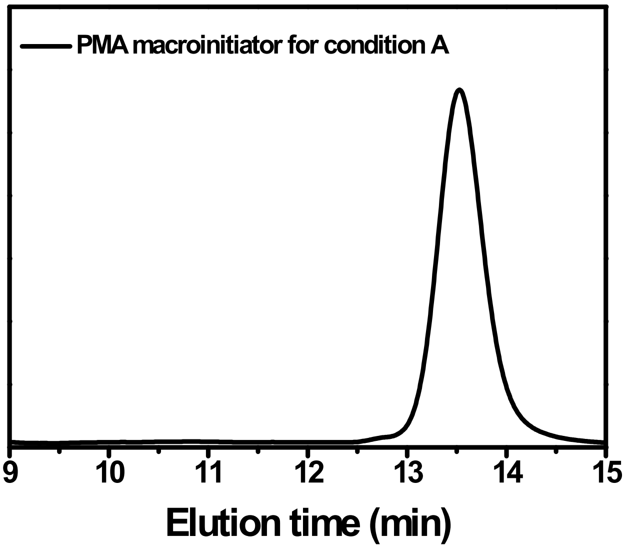

Figure 17S. PMA macroinitiator for irradiation with Xe lamp, $[MA]_0/[\mathbf{I}]_0 = 600/1$, I = 3 mw/cm^2, room temperature, t = 10h, conversion = 26%, $M_{n,GPC}$ = 13600, M_w/M_n = 1.07.

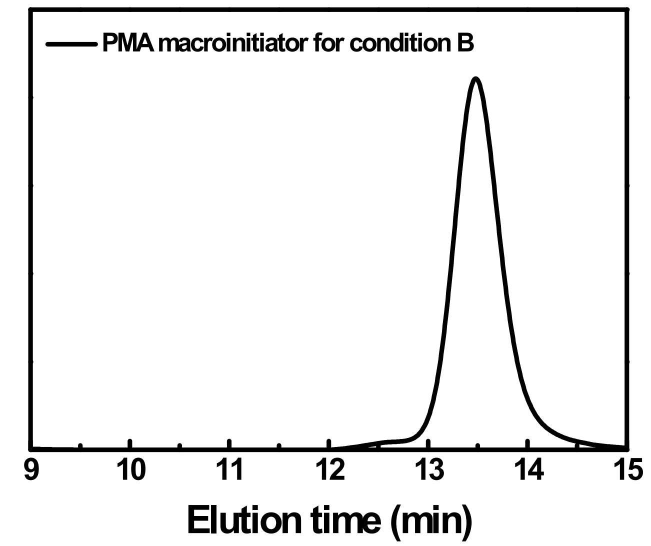

Figure 18S. PMA macroinitiator for irradiation with CFL lamp, $[MA]_0/[\mathbf{I}]_0 = 600/1$, $I = 3\text{-}5$ mW/cm^2, room temperature, t = 12.5h, conversion = 26%, $M_{n,\ GPC} = 14100$, $M_w/M_n = 1.07$.

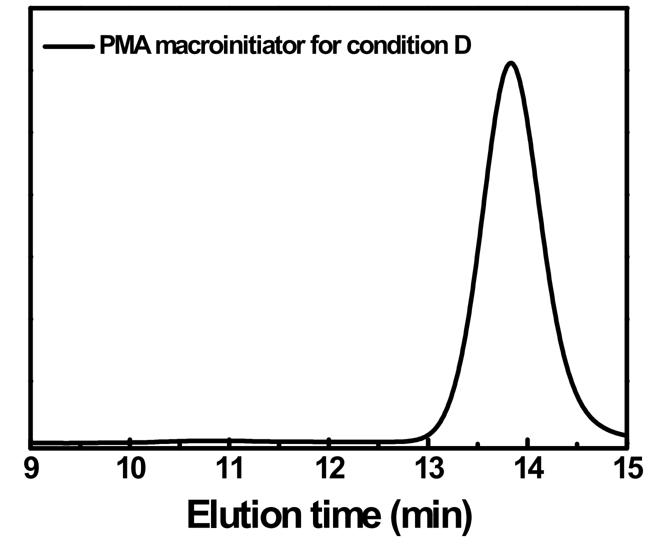

Figure 19S. PMA macroinitiator for irradiation with Xe lamp with addition of 1 equivalent of TPO, $[M]_0/[\mathbf{I}]_0/[TPO]_0 = 600/1/1$, $I = 3$ mw/cm^2, room temperature, t = 1.5h, conversion = 23%, $M_{n,\ GPC} = 9700$, $M_w/M_n = 1.11$.

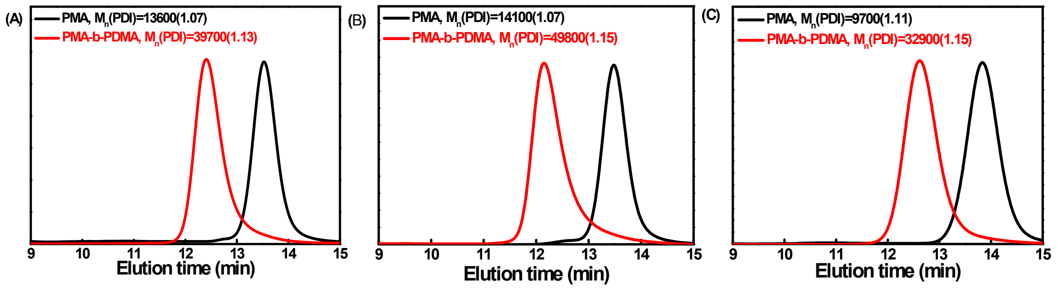

Figure 20S. GPC traces of block copolymer PMA-b-PDMA (black line = macroinitiator, red line = block copolymer) with $[DMA]_0$ = 1.0 M, $[DMA]_0/[(salen)Co\text{-}PMA]_0$ = 600/1: (A) irradiated by Xe lamp, t = 12 h, conv. = 54%, $M_{n,GPC}$ = 39700, M_w/M_n = 1.13; (B) irradiated by CFL, t = 18 h, conv. = 51%, $M_{n,GPC}$ = 49800, M_w/M_n = 1.15; (C) irradiated by Xe lamp with addition of 1 equivalent of TPO, t = 2 h, conversion = 36%, $M_{n,GPC}$ = 32900, M_w/M_n = 1.15.
